# A Genome-Wide Association Study Pinpoints Quantitative Trait Genes for Plant Height, Heading Date, Grain Quality, and Yield in Rye (*Secale cereale* L.)

**DOI:** 10.3389/fpls.2021.718081

**Published:** 2021-10-29

**Authors:** Dörthe Siekmann, Gisela Jansen, Anne Zaar, Andrzej Kilian, Franz Joachim Fromme, Bernd Hackauf

**Affiliations:** ^1^Julius Kühn Institute, Federal Research Centre for Cultivated Plants, Institute for Breeding Research on Agricultural Crops, Sanitz, Germany; ^2^HYBRO Saatzucht GmbH & Co. KG, Schenkenberg, Germany; ^3^Julius Kühn Institute, Federal Research Centre for Cultivated Plants, Institute for Resistance Research and Stress Tolerance, Sanitz, Germany; ^4^Diversity Arrays Technology, Bruce, ACT, Australia

**Keywords:** phenotyping, drought stress, DNA profiling, SNP, tiller number, SMART breeding, WOX transcription factor, arabinoxylan (AX)

## Abstract

Rye is the only cross-pollinating Triticeae crop species. Knowledge of rye genes controlling complex-inherited traits is scarce, which, currently, largely disables the genomics assisted introgression of untapped genetic variation from self-incompatible germplasm collections in elite inbred lines for hybrid breeding. We report on the first genome-wide association study (GWAS) in rye based on the phenotypic evaluation of 526 experimental hybrids for plant height, heading date, grain quality, and yield in 2 years and up to 19 environments. We established a cross-validated NIRS calibration model as a fast, effective, and robust analytical method to determine grain quality parameters. We observed phenotypic plasticity in plant height and tiller number as a resource use strategy of rye under drought and identified increased grain arabinoxylan content as a striking phenotype in osmotically stressed rye. We used DArTseq™ as a genotyping-by-sequencing technology to reduce the complexity of the rye genome. We established a novel high-density genetic linkage map that describes the position of almost 19k markers and that allowed us to estimate a low genome-wide LD based on the assessed genetic diversity in elite germplasm. We analyzed the relationship between plant height, heading date, agronomic, as well as grain quality traits, and genotype based on 20k novel single-nucleotide polymorphism markers. In addition, we integrated the DArTseq™ markers in the recently established ‘Lo7' reference genome assembly. We identified cross-validated SNPs in ‘Lo7' protein-coding genes associated with all traits studied. These include associations of the WUSCHEL-related homeobox transcription factor *DWT1* and grain yield, the DELLA protein gene *SLR1* and heading date, the Ethylene overproducer 1-like protein gene *ETOL1* and thousand-grain weight, protein and starch content, as well as the Lectin receptor kinase *SIT2* and plant height. A Leucine-rich repeat receptor protein kinase and a Xyloglucan alpha-1,6-xylosyltransferase count among the cross-validated genes associated with water-extractable arabinoxylan content. This study demonstrates the power of GWAS, hybrid breeding, and the reference genome sequence in rye genetics research to dissect and identify the function of genes shaping genetic diversity in agronomic and grain quality traits of rye. The described links between genetic causes and phenotypic variation will accelerate genomics-enabled rye improvement.

## Introduction

Rye (*Secale cereale* L.) is the only allogamous crop in the Triticeae tribe of the grasses. Natural genetic diversity in outbreeding rye enabled to achieve a series of technological advances that ultimately facilitated the establishment of hybrid breeding (Hackauf et al., 2021), a key technology for increasing and securing cereal production on finite arable land without increasing water and fertilizer use (Whitford et al., 2013). Hybrid breeding resulted in a strong response to the selection of favorable alleles for grain and quality traits in rye (Laidig et al., 2017) and contributed to keeping this orphan crop competitive in modern agricultural production systems. ‘Petkus' and ‘Carsten' represent two major germplasm pools exploited in hybrid rye breeding (Geiger and Miedaner, 2009). Beyond heterotic groups and just like in maize (White et al., 2020), the structure of commercial hybrid rye breeding is characterized by the largely isolated and unique sub-heterotic patterns of major breeding programs (Bauer et al., 2017; Vendelbo et al., 2020). As elite germplasm utilization across programs is impossible, capture and management of genetic diversity from germplasm resources are of outstanding importance for the long-term success of commercial hybrid rye breeding programs. However, the targeted identification of novel genetic variation in agronomically important genes, especially superior alleles occurring with low frequencies in self-incompatible rye germplasm collections, is currently largely disabled. This accounts for the fact that comparable progress in gene discovery like in barley (Pasam et al., 2012; Hill et al., 2021; Li M. et al., 2021) or wheat (Liu et al., 2017; Gao et al., 2021) has yet not been achieved in rye. With the recent release of two high-quality genome assemblies (Li G. et al., 2021; Rabanus-Wallace et al., 2021), rye has finally reached the genome era, enabling the integration and advancement of fundamental and applied breeding and research to understand how the genome builds, maintains, and operates rye. In order to accelerate the transition from merely phenotypic to haplotype-based breeding (Bevan et al., 2017; Brinton et al., 2020) and to substantially increase the efficiency, precision, and flexibility of rye breeding, further progress in rye genomic research is necessary to associate genome sequence information with phenotypes related to rye growth and development. This is particularly relevant to grain quality parameters, as the versatile uses of rye in the production of bread or mixed animal feeds have, so far, been considered to require highly divergent breeding goals (Kobylyansky et al., 2019). An efficient selection of grain quality parameters, particularly with respect to the content of arabinoxylans as the predominant dietary fiber in the rye grain, is currently a limiting factor in rye breeding.

Grain quality, as well as agronomic important traits controlling plant height, heading date, thousand-grain weight, or yield, reveal a continuous phenotypic variation and are genetically controlled by a network of multiple and interacting loci (Mackay et al., 2009). In rice, cloning of these quantitative trait loci (QTL) for grain yield components and other agronomic important traits (Yonemaru et al., 2010; Yamamoto et al., 2012; Li et al., 2018) has a significant impact on the genetic improvement of this important staple food (Xing and Zhang, 2010; Wang and Li, 2019). In contrast, comprehensive QTL analysis in interpool rye hybrids with a high heterosis level currently refers to a single biparental mapping population from the ‘Carsten' gene pool crossed to a CMS tester from the ‘Petkus' gene pool (Hackauf et al., 2017a; Miedaner et al., 2018). Compared to QTL mapping with biparental populations, genome-wide association studies (GWAS) offer a sampling of greater levels of genetic diversity and higher resolution of QTL positions using already existing breeding lines and genetic stocks (Jamann et al., 2015). In rye, GWAS have been reported for the traits leaf rust resistance, pre-harvest sprouting resistance, and α-amylase activity (Rakoczy-Trojanowska et al., 2017), Tan spot resistance (Sidhu et al., 2019), Fusarium head blight resistance (Gaikpa et al., 2020), and stem rust resistance (Gruner et al., 2021).

Here, we describe the first comprehensive GWAS for plant architecture, grain quality, and yield in rye. We aimed to (i) assess the genetic variation for plant height, heading date, tiller number, grain yield, grain weight, grain protein, and starch, as well as total and water-extractable arabinoxylan content in rye, (ii) identify QTL for these traits by GWA mapping and estimate their effects, (iii) investigate the co-localization between QTL for grain yield and yield parameters, as well as between grain quality parameters. Furthermore, we used the recently released ‘Lo7' genome assembly (Rabanus-Wallace et al., 2021) to describe the genetic architecture of the assessed traits.

## Materials and Methods

### Plant Materials

The genetic materials analyzed in this study encompass two data sets of advanced-cycle inbred lines of a commercial hybrid rye breeding program. The two data sets represented a gametic sample of both heterotic gene pools and comprised a total of 126 S5-lines of the ‘Petkus' and 15 restorer synthetics of the ‘Carsten' gene pool. Male-sterile BC2-analogs of S5-lines from the ‘Petkus' pool have been developed by backcrossing into the CMS-inducing Pampa (P) cytoplasm (Geiger and Schnell, 1970). We have used the line x tester mating design of Kempthorne (1956) to develop two large multiple-hybrid populations using the male-sterile BC2-analogs as females to be tested and the restorer synthetics as testers. A total of 298 top cross hybrids were obtained in the first data set by pollinating 79 BC2-analogs from the ‘Petkus' pool with 12 synthetics from the ‘Carsten' pool. In the second data set, 305 top cross hybrids originated from 84 BC2-analogs from the ‘Petkus' pool pollinated by 11 synthetics from the ‘Carsten' pool. Both data sets were connected by 77 common experimental hybrids. These plant materials are proprietary to HYBRO Saatzucht GmbH & Co. KG. The released hybrid varieties ‘Rasant', ‘Askari', ‘Minello', ‘Brasetto', ‘Palazzo', ‘Visello', as well as ‘SU Mephisto', ‘Palazzo', ‘Visello', ‘Brasetto', ‘SU Allawi', and ‘Minello', were used as checks in 2011 and 2012, respectively.

### Phenotypic Data Analyses

Experimental hybrids of data set 1 were evaluated in 2011 at 10 locations, representing target environments of rye cultivation in Germany and Poland. Soils with different qualities in agricultural terms enabled to define two locations each at the sites Kleptow and Wulfsode as measured on a scale of soil value of arable land (German: Bodenwertzahl), which is determined from soil sampling data and that ranges from 0 (very low) to 100 (very high). The soil type at Kleptow is a loam, partly with loess covering (soil value 60), while Kleptow-Sand is a heavy-to-clayey loam (soil value 45), just like at Wulfsode (soil value 38). The soil at Wulfsode-Sand is a sandy loam (soil value 25). Thus, the locations comprise (1) Kleptow (KLE) N53.4°, E13.9°, (2) Kleptow-Sand (KLS) N53.4°, E13.9°, (3) Wulfsode (WUL) N53°, E10.2°, (4) Wulfsode-Sand (WLS) N53°, E10.2° (5) Cappeln (WES) N52.8°, E8°, (6) Bornhof (BOH) N53.5°, E12.9°, (7) Granskevitz (GKV) N54.5°, E13.2°, (8) Sulejów (SUL) N51.4°, E19.8°, (9) Poznań (POZ) N52.4°, E16.6°, and (10) Uhnin (UHN) N51.6°, E23°. In 2012, the location WLS was not included. The location × year combinations are subsequently referred to as environments. The year 2011 and, in particular, the period from January to May was characterized by natural drought stress in Europe with many areas receiving <40% of long-term mean annual precipitation [DWD (Deutscher Wetterdienst), 2012]. In contrast, no precipitation anomalies in relation to the long-term mean for 1951–2000 have been recorded for the hydrological period from November 2011 until May 2012 in the North German Plain and Poland [DWD (Deutscher Wetterdienst), 2012]. Entries were allocated to trials laid out as α-lattice designs with two replicates on 5 to 6 m^2^ plots, connected by elite hybrid checks. Test cross performance was evaluated for grain yield (GYD, Mg ha^−1^), plant height (PHT, cm), thousand-grain weight (TGW, g), heading date (HDT, 1 = late – 9 = early), tiller number (TIN), grain protein (GPC, g/kg), and starch (STC, g/kg), as well as total arabinoxylan (TAX, g/kg), and water-extractable arabinoxylan (WAX, g/kg) content. For TGW data across 10 environments (KLE2011, KLS2011, BOH2011, WLS2011, POZ2011, KLE2012, KLS2012, BOH2012, WUL2012, and WES2012) were recorded. GPC, STC, TAX, and WAX were assessed in eight environments (KLE2011, KLS2011, BOH2011, WLS2011, POZ2011, KLS2012, KLE2012, and WUL2012), while TIN was assessed in three environments (KLE2011, KLS2011, and KLE2012). Grain yield was adjusted to a moisture content of 140 g H_2_O kg^−1^. We have used a near-infrared spectroscopy (NIRS) calibration (Agelet and Hurburgh, 2010) to predict grain quality parameters in experimental rye hybrids. This NIRS calibration was adjusted in both data sets by grain samples selected from the experimental hybrids of both years. Subsequent to non-destructive NIRS scans, the content of GPC, TAX, as well as WAX, was assayed as recently described (Jürgens et al., 2012). Native starch content was determined according to the Ewers polarimetric method (ISO 10520) using a Polartronic universal polarimeter (Schmidt + Haensch, Berlin, Germany).

For each data set, best linear unbiased estimators (BLUEs) of the experimental hybrids were calculated over locations according to model Equation (1):


(1) $$\begin{array}{r} y_{ijkl}=\mu +g_{i}+\;\alpha_{j}+\;\delta_{ij}+\;\beta_{jk}+\;\varphi_{jkl}+\;\varepsilon_{ijkl} \end{array}$$

where *y*_*ijkl*_ is the phenotypic observation of the *i*th hybrid at the *j*th location in the *l*th in complete block of the *k*th replication; μ, the intercept; *g*_*i*_, the genetic effect of the *i*th hybrid; α_*j*_, the effect of the *j*th location; δ_*ij*_, the genotype-by-environment interaction; β_*jk*_, the effect of the *k*th replicate at the *j*th location; φ_*jkl*_, the effect of the *l*th incomplete block of the *k*th replication at the *j*th location; and ε_*ijkl*_, the residual. μ and *g*_*i*_ were regarded as fixed, all other effects as random. For estimation of variance components, *g*_*i*_ was defined as random. Broad-sense (entry-mean) heritability (H^2^) was estimated based on Equation (2):


(2) $$\begin{array}{r} H^{2}=\;\frac{\sigma_{G}^{2}}{\sigma_{G}^{2}+\frac{\sigma_{GxE}^{2}}{E}+\frac{\sigma_{e}^{2}}{ER}} \end{array}$$

with $\sigma_{\text{G}}^{2}$, $\sigma_{\text{GxE}}^{2}$, and $\sigma_{\text{e}}^{2}$ being the variances of genotype, genotype-by-environment interaction, and residual plot error, respectively. E denotes the number of environments and R the number of replications per environment. Analysis of phenotypic data was performed using package lme4 in R (R Core Team, 2017). Estimation of variance components was conducted using the REML procedure in R. Pearson's correlation coefficients between phenotypic traits were calculated based on BLUEs with R package metan and visualized using package pheatmap in R.

### Genotyping and Linkage Map Construction

DNA profiles were established for a panel of 180 ‘Petkus' and 34 ‘Carsten' genotypes as well as 91 plants of the RIL-population L2039-N x DH (Martis et al., 2013; Hackauf et al., 2017a). DNA samples were genotyped using DArT™ and DArTseq™-technology as previously described (Bolibok-Bragoszewska et al., 2009; Targońska-Karasek et al., 2017) at Diversity Arrays Technology Pty. Ltd., Yarralumla ACT, Australia. Marker genotypes of the single cross hybrids were inferred from the parental genotypes. For genetic map construction, markers with more than 10% missing values and more than 5% heterozygote genotypes were excluded from the analysis. Perfectly identical marker loci were identified in JoinMap 4 (Van Ooijen, 2006) and removed from the core data set to reduce calculation efforts. Markers with a maximum of 1% missing values were used to establish a framework map, defining the number, joint genotype, and position of genomic bins. Grouping of DArTseq™ markers, together with DArT™, single-nucleotide polymorphism (SNP), and SSR markers, from the L2039-N x DH map (Martis et al., 2013) was conducted using QTL IciMapping 3.2 (Meng et al., 2015). The high-density linkage map was constructed using the locus-ordering algorithm RECORD (Van Os et al., 2005). Map quality was evaluated by computing the sum of adjacent recombination fractions (SARF) with a window size of 5. DArTseq™ SNP tags were integrated into the ‘Lo7' genome assembly (Rabanus-Wallace et al., 2021) by using the Basic Local Alignment Search Tool (BLAST) (Altschul et al., 1990). A significance threshold with more than 98% identity of at least 65 bp was used. Cloned and functionally characterized rice genes (Yonemaru et al., 2010) were queried by BLASTP against the ‘Lo7' peptide sequences (Rabanus-Wallace et al., 2021) with an E-value < 1e-20 as a significance threshold. BLASTN sequence similarity searches were conducted as described (Hackauf et al., 2009) to integrate first-generation DArT™ as well as EST-derived markers flanking previously identified QTL (Hackauf et al., 2017a; Miedaner et al., 2018) in the ‘Lo7' genome assembly.

### Analysis of Genetic Diversity, Linkage Disequilibrium, Kinship, Principal Coordinates, and Population Structure

For the molecular characterization of elite rye inbred lines, SNPs with a call rate > 0.8 and a minor allele frequency (MAF) > 0.05 were selected using the R package snpReady. The basic population genetic statistics observed heterozygosity (*H*_*obs*_), gene diversity (expected heterozygosity, Ĥ, Nei, 1987), inbreeding coefficient (*F*_*IS*_), as well as the Analysis of Molecular Variance (AMOVA, Excoffier et al., 1992), were calculated using the R package hierfstat. A Cavalli-Sforza and Edwards (1967) chord distance matrix was calculated with the R package hierfstat and analyzed in a Principal Coordinates Analysis (PCoA) as well as hierarchical clustering using the Unweighted Pair Group Method with Arithmetic mean (UPGMA) algorithm using the R packages ape and stats, respectively. The genetic differentiation between both heterotic gene pools was inferred based on the pairwise *F*_*ST*_ estimator proposed by (Weir and Cockerham, 1984). For evaluation of linkage disequilibrium (LD) in the two phenotypic data sets, mapped SNP markers with MAF > 5% were used to calculate squared allele frequency correlation (${\text{r}}_{\text{vs}}^{2}$) values with the R package LDcorSV. To correct the estimation of LD for relatedness between the single hybrids, kinship coefficients were calculated with dominant DArT™ and SilicoDArT™ markers according to Hardy (2003) using the software SPAGeDi (Hardy and Vekemans, 2002). To eliminate negative kinship values while keeping variation, all coefficients were adjusted for the lowest value instead of setting all negative coefficients to zero. For analysis of population structure, five independent runs were performed for K = 1–9 using 1,523 dominant DArT™ markers in data set 1 and 4,000 randomly chosen SilicoDArT™ markers in data set 2, with the software Structure v2.2.3 Pritchard et al. (2000), with a burn-in of 100,000 and 200,000 iterations. The probable number of populations was determined according to Evanno et al. (2005). Both factors, kinship and population structure, were taken into account during the calculation of ${\text{r}}_{\text{vs}}^{2}$ values. Non-linear regression was used to analyze the decay of LD with genetic distance for each chromosome separately (Remington et al., 2001). A critical value for the determination of LD decay was calculated in R from the distribution of square root-transformed ${\text{r}}_{\text{vs}}^{2}$ values of the unlinked loci. The 95th percentile of that distribution served as the threshold value, beyond which LD was likely to be caused by genetic linkage. Visualization of LD calculation results was done with the R package LDheatmap. For PCoA in the two sets of experimental hybrids, polymorphic SilicoDArT™ markers with a call rate > 0.95 were used to calculate Cavalli-Sforza and Edwards (1967) chord distance matrices. All visualizations were done with the packages ggplot2 and ggtree in R.

### Genome-Wide Association Mapping

Single-nucleotide polymorphism markers were filtered for MAF > 5% and a call rate > 0.2 in TASSEL 3 (Bradbury et al., 2007) to identify marker/trait associations in the two data sets. Taking the cofactors kinship and population structure into account, a mixed linear model approach according to model Equation (3) was used in combination with the P3D algorithm (“population parameters previously determined”) to determine significant associations in TASSEL:


(3) $$\begin{array}{r} y=X\beta +Zu+e \end{array}$$

where *y* is the vector of observations; β represents an unknown vector containing fixed effects, including genetic marker and population structure; u is an unknown vector of random additive genetic effects from multiple-background QTL for individuals; X and Z are the known design matrices; and e is the unobserved vector of random residual. Vector u and e are assumed to be normally distributed with null mean and variance of $Var\;\begin{array}{c} u\\ e \end{array}=\;\left(\begin{array}{cc} G & 0\\ 0 & R \end{array}\right)$, where G = $\sigma_{\text{a}}^{2}$K with $\sigma_{\text{a}}^{2}$ as additive genetic variance and K as kinship matrix. R is the residual effect with R = I $\text{σ}{\;}_{e}^{2}$, where $\text{σ}{\;}_{e}^{2}$ is the residual variance.

To adjust raw *p*-values for multiple hypotheses testing, the false discovery rate was controlled according to Benjamini and Hochberg (1995) using the R package multtest. The localization of associated SNPs was illustrated using MapChart (Voorrips, 2002). Phenotypic variances (R^2^) explained by individual SNPs were plotted using ggplot2 in R.

## Results

### Elite Rye Germplasm Reveals Pronounced Genetic Variation in Agronomic and Quality Traits

**Figure 1:**
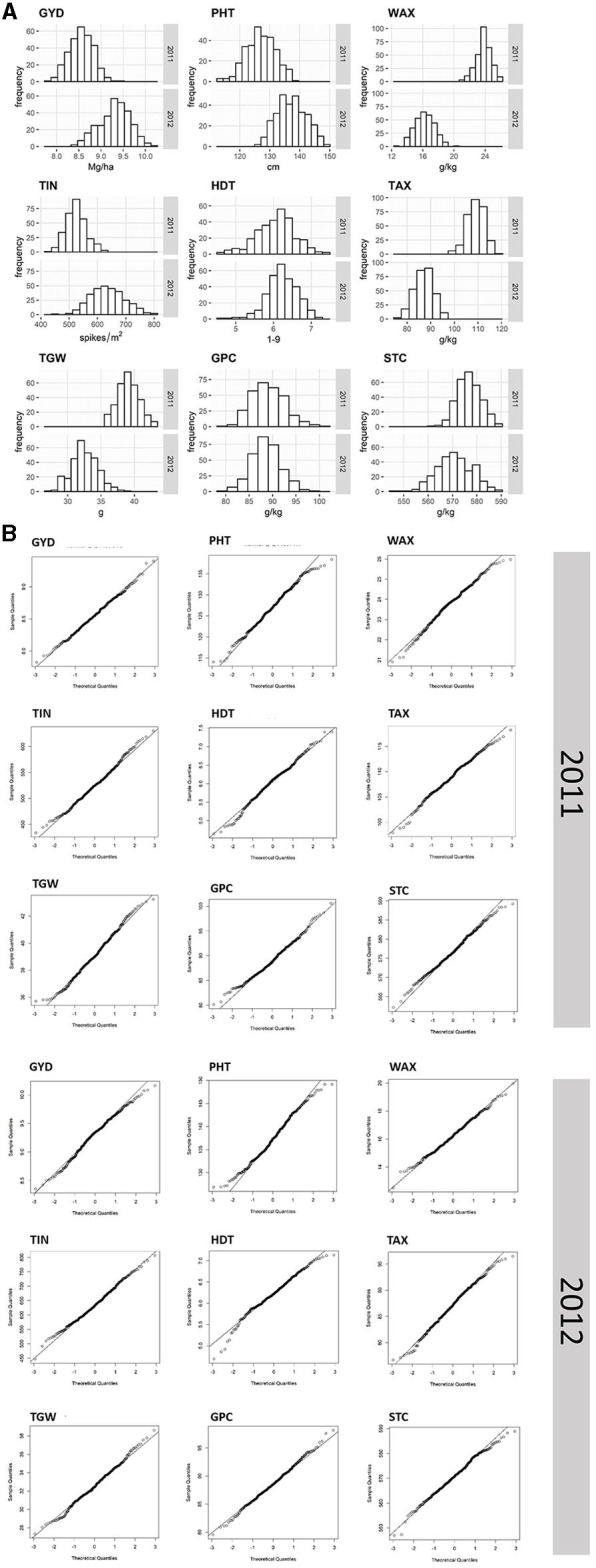
Frequency distribution histograms **(A)** and Quantile-Quantile (Q-Q) plots **(B)** of nine agronomic and quality traits evaluated for 298 and 305 single cross rye hybrids in 2011 and 2012. The vertical axes in the histograms indicate the frequency of hybrids per trait (GYD, grain yield; TIN, tiller number; TGW, thousand-grain weight; PHT, plant height; HDT, heading date; GPC, grain protein content; WAX, water extractable arabinoxylan content; TAX, total arabinoxylan content; STC, starch content) and the horizontal axes indicate the different trait classes.

Analysis of variance (ANOVA) revealed that genotypic effects were statistically significant (*p* < 0.05) for all traits except TIN in 2011 (Table 1). All traits but GPC, STC, WAX, and TAX in 2012 as well as TIN showed significant (*p* < 0.05) genotype x environment (GxE) interaction variances. The GxE interaction variances of GYD in both years, as well as of WAX in 2011 and HDT in 2012, were larger than the variances of the genotype (Table 1). The average performance in grain yield (−8%), TIN (−17%), and PHT (−8%) was lower in 2011 as compared with 2012. In contrast, the average TGW (+20%), as well as the content of TAX (+25%) and WAX (+47%), was higher in 2011 as compared with 2012. High heritability estimates depict a low error variance and a high genetic variance for all traits with the exception of TIN (H^2^ = 0.3). Q-Q plots are fairly straight and indicate that normal distribution is a reasonably good approximation for all traits (Figure 1).

**Table 1:** First and second order statistics of experimental rye hybrids.

**Year**	**2011**						**2012**					
**Trait**	**μ ± s**	**x_**min**_-x_**max**_**	** $\sigma_{\text{G}}^{2}$ **	** $\sigma_{\text{GxE}}^{2}$ **	** $\sigma_{\text{e}}^{2}$ **	**H^**2**^**	**μ ± s**	**x_**min**_-x_**max**_**	** $\sigma_{\text{G}}^{2}$ **	** $\sigma_{\text{GxE}}^{2}$ **	** $\sigma_{\text{e}}^{2}$ **	**H^**2**^**
GYD [Mg/ha]	8.56 ± 0.28	7.82–9.4	0.05*	0.13*	0.18	0.69	9.31 ± 0.35	8.35–10.16	0.09*	0.23*	0.16	0.71
PHT [cm]	126.9 ± 4.9	114.1–138.4	22.03*	7.14*	13.92	0.94	137.5 ± 4.8	126.8–149.1	21.65*	5.23*	13.9	0.94
HDT [1–9]	6.03 ± 0.51	4.65–7.4	0.22*	0.18*	0.32	0.87	6.23 ± 0.4	4.69–7.14	0.11*	0.26*	0.3	0.72
TIN [spikes/m^2^]	526.64 ± 35.6	423.5–630.2	339.4	0	2926.8	0.32	637.63 ± 60.5	446.8–806.8	898.3*	NA	3734.6	0.33
TGW [g]	39.1 ± 1.6	35.7–43.3	2.19*	0.87*	1.22	0.88	32.55 ± 1.96	27.3–38.6	3.47*	0.96*	1.42	0.91
GPC [g/kg]	89.4 ± 3.48	80.1–100.6	10.1*	2.04*	10.3	0.88	88.96 ± 3.1	79.6–98.2	6.2*	1.2	11.3	0.73
STC [g/kg]	576.7 ± 4.86	562.2–589.2	20.2*	5.07*	20.2	0.87	570.7 ± 7.3	546.9–589	31.1*	1.8	99.6	0.64
WAX [g/kg]	23.9 ± 1	20.9–27	0.73*	0.81*	0.79	0.75	16.3 ± 1.2	12.5–20	0.87*	0	2.7	0.66
TAX [g/kg]	109.1 ± 3.7	98–119.1	12.1 *	4.2*	6.8	0.89	87.1 ± 3.9	76.7–96.5	12.2*	1.8	12.2	0.82

Means and standard deviation (μ ± s), minimum and maximum (x_min_-x_max_), estimates of variance components (genotype, $\sigma_{\text{G}}^{2}$; genotype x environment interaction, $\sigma_{\text{GxE}}^{2}$; error, $\sigma_{\text{e}}^{2}$), and entry-mean heritabilities (H^2^) for agronomic and quality traits evaluated across 3–19 environments in 2011 and 2012. GYD, grain yield; PHT, plant height; HDT, heading date; TIN, tiller number; TGW, thousand-grain weight; GPC, grain protein content; STC, starch content; TAX, total arabinoxylan content; WAX, water-extractable arabinoxylan content. *p = 0.05.

**Figure 2:**
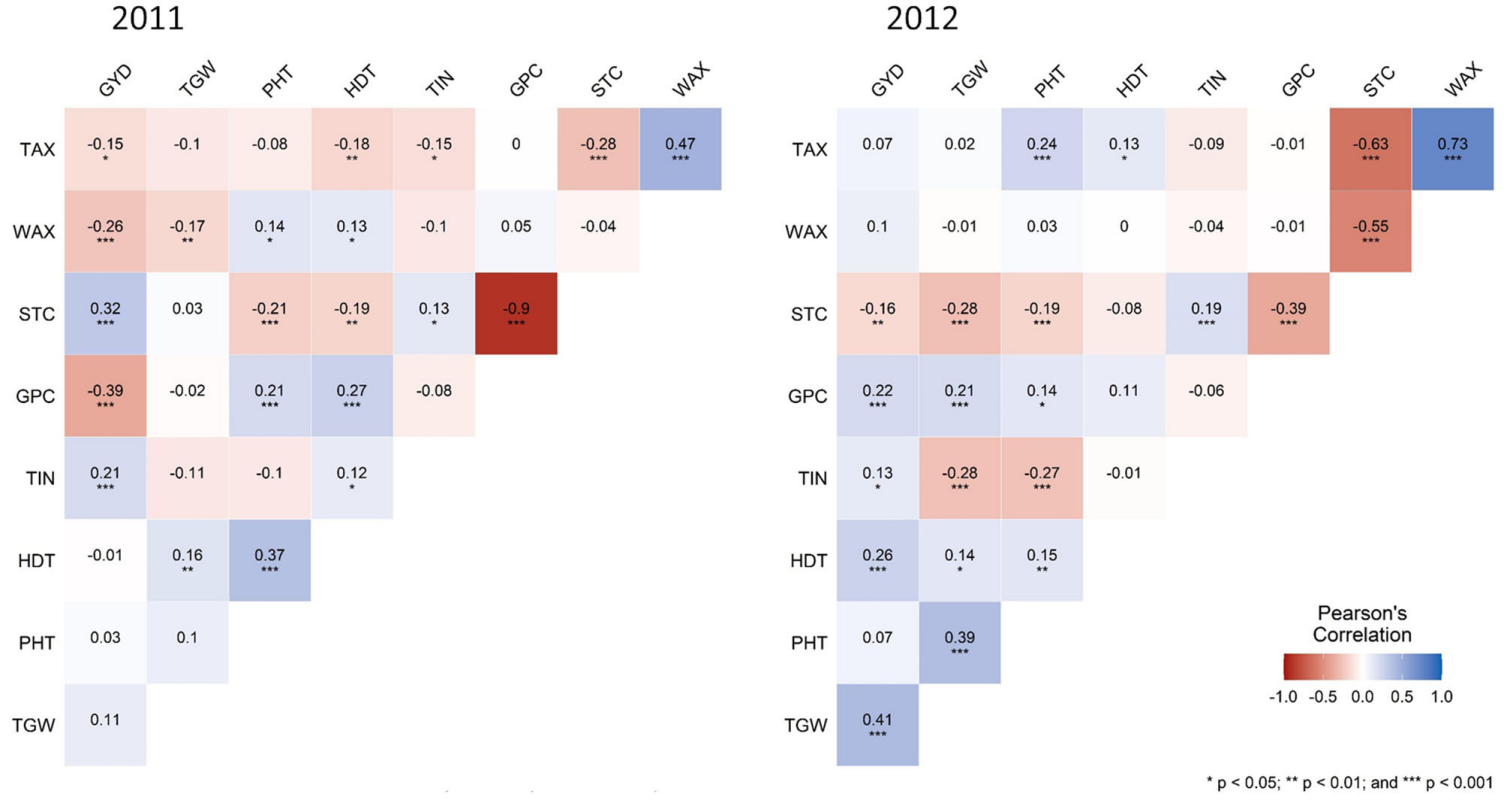
Heatmaps of Pearson's correlation coefficients for nine agronomic and quality traits in rye. Blue color indicates positive correlation and red color indicates negative correlation among different traits, with color intensity variance depicting the strength of correlation. GYD, grain yield; TIN, tiller number; TGW, thousand-grain weight; PHT, plant height; HDT, heading date; GPC, grain protein content; WAX, water extractable arabinoxylan content; TAX, total arabinoxylan content; STC, starch content. Categorization: 0.25 ≤ r <0.45 weak, 0.45 ≤ r < 0.65 moderate, 0.65 ≤ r < 0.85 strong, 0.85 ≤ r very strong. *p < 0.05, **p < 0.01, ***p < 0.001, blank for non-significant.

The pattern of phenotypic correlation coefficients is illustrated in Figure 2. Most significant (*p* < 0.001) and consistent correlations were found between STC and PHT, STC and GPC, STC and TAX, as well as between TAX and WAX. The significant (*p* < 0.01) correlations between GYD and STC as well as GPC are in opposite directions in both years. GYD, TGW, and HDT, as well as TGW, STC, GPC, TIN, and PHT, revealed significant (*p* < 0.001) correlations in 2012 only. Similarly, the correlation between STC and WAX was significant (*p* < 0.001) in 2012 only. Plant height was significantly (*p* < 0.001) correlated with TGW in 2012 and starch content in both years but neither correlated with GYD in 2011 nor in 2012. A very strong negative correlation (r = −0.9) could be observed between GPC and STC in 2011 only. No moderate correlations were found except the correlations previously mentioned (Figure 2).

### Significant Molecular Differentiation of Elite Rye Germplasm

**Figure 3:**
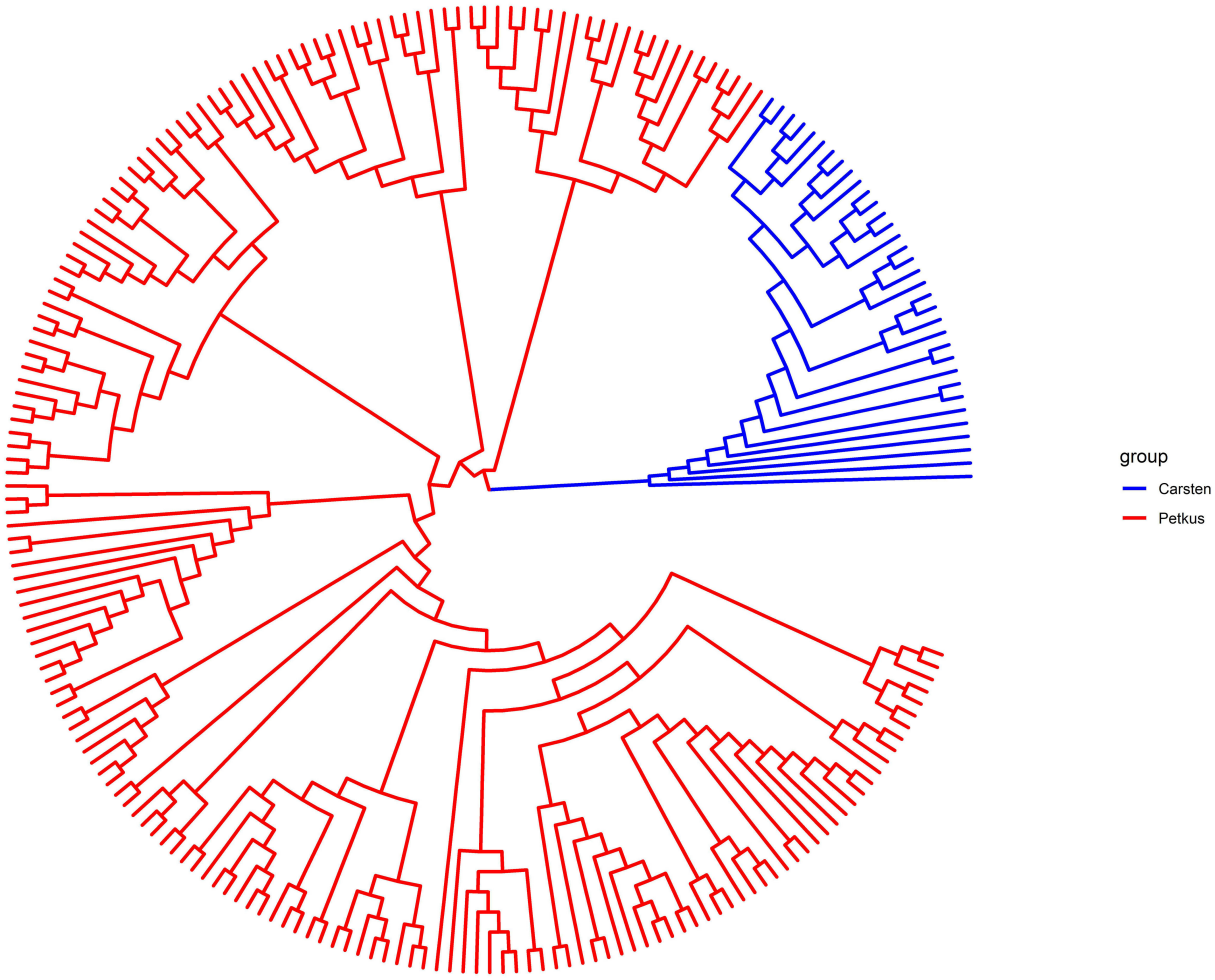
UPGMA-based dendrogram showing the genetic relationship among the 214 elite seed and pollen parent lines based on 2,965 SNP markers. Samples are colored according to the heterotic gene pools ‘Petkus' (seed parent pool) and ‘Carsten' (pollen parent pool).

**Figure 4:**
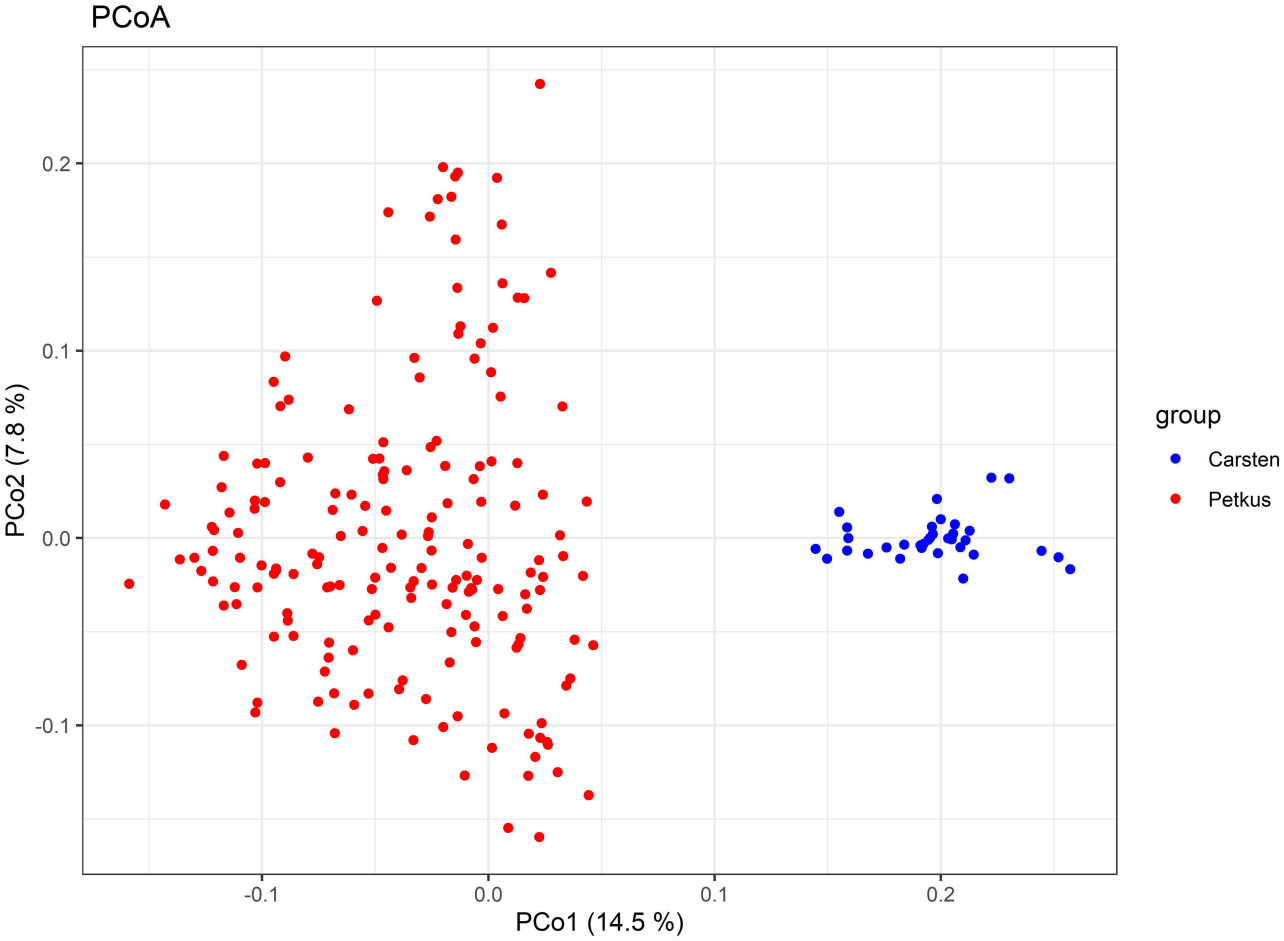
Principal coordinates analysis (PCoA) of 214 elite seed and pollen parent lines. Scatter plot of the first two principal coordinates based on genotyping data of 2,965 SNP markers. The horizontal and vertical coordinates represent PCo1 and PCo2. Each dot represents a line. Samples are colored according to the heterotic gene pools ‘Petkus' (seed parent pool) and ‘Carsten' (pollen parent pool).

A total of 2,965 SNPs were successfully called with high quality in 214 seed and pollen parent lines. In total, 2,006 of these SNPs (67.7%) could be mapped in the ‘Lo7' reference genome sequence and revealed an equal distribution on the seven ‘Lo7' pseudomolecules. The average PIC of these SNPs was 0.258, ranging from 0.09 to 0.38. A detailed list of these informative SNP loci, including physical map position in the ‘Lo7' genome assembly, base change, MAF, heterozygosity, gene diversity, and PIC, is provided in Supplementary Table 1. The average heterozygosity of each ‘Petkus' line was 6%, while, for the ‘Carsten' genotypes, an average heterozygosity of 29% (Table 2) was determined. Analysis of Molecular Variance (AMOVA) results revealed that molecular variation was mainly (57.1%) found among individuals within populations as expected for cross-pollinated species, whereas variation observed among populations within groups explained 18.5% of the total genetic variability (Table 3). The calculated fixation index *F*_*ST*_= 0.185 of Weir and Cockerham (1984) emphasized a significant differentiation between both heterotic populations. Cavalli-Sforza and Edwards (1967) chord genetic distances between pairwise comparisons of all the 214 lines ranged from 0.012 to 0.487, and the overall average distance was 0.356. The UPGMA tree clustered this population into two major groups, perfectly in line with the well-known heterotic groups in rye (Figure 3). The major group was composed solely of ‘Petkus' lines and was divided into five clades comprising 18–84 lines each. In the second monophyletic branch, all ‘Carsten' lines are grouped (Figure 3). PCoA on the entire set of 214 lines (Figure 4) showed a clear separation of the two groups identified in the cluster analysis. The first two principal coordinates (PCos) from PCoA explained 22.3% of the total SNP variation among the samples.

**Table 2:** Genetic diversity of elite rye inbred lines representing the heterotic gene pools ‘Petkus' and ‘Carsten'.

**Population name**	**Breeding level**	**Individuals**	***A_***p***_***	***H_***obs***_***	***H***	***F_***IS***_***
‘Petkus'	Elite seed parent	180	247	0.06	0.30	0.77
‘Carsten'	Elite pollen parent	34	26	0.29	0.31	0.07

*The level of genetic diversity of each population was estimated based on 2,965 SNP markers and described with the parameters number of private alleles (A_p_), observed heterozygosity (H_obs_), gene diversity (expected heterozygosity, H), and inbreeding coefficient (F_IS_)*.

**Table 3:** Analysis of molecular variance (AMOVA) for the total breeding population.

**Source of variation**	**Variance component**	**Proportion of explained variation**
Among populations	203.26	0.185
Within populations	627.30	0.571
Within individuals	268.33	0.244
Total	1098.89	

### Family Substructure and Rapid Decay of LD in Experimental Rye Hybrids

**Figure 5:**
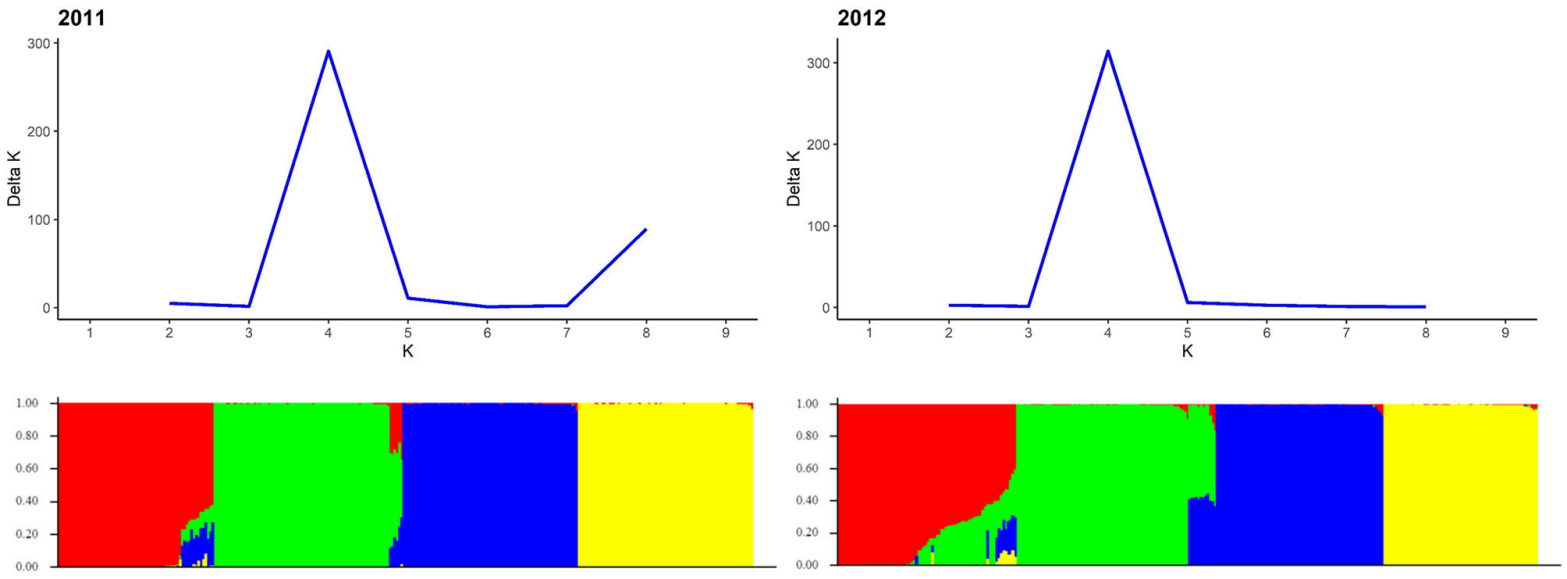
Population structure based on genotyping data of 1,523 and 4,000 SilicoDArT™ markers for 298 and 305 single cross rye hybrids in 2011 and 2012, respectively. Each genotype is represented by a thin vertical line, which is partitioned into k = 4 colored segments that represent the genotype's estimated membership fractions shown on the y-axis in k clusters. Genotypes were sorted according to populations along the x-axis. Determination of the most probable number of k-groups according to Evanno et al. (2005) is displayed in the upper part of the graph.

**Figure 6:**
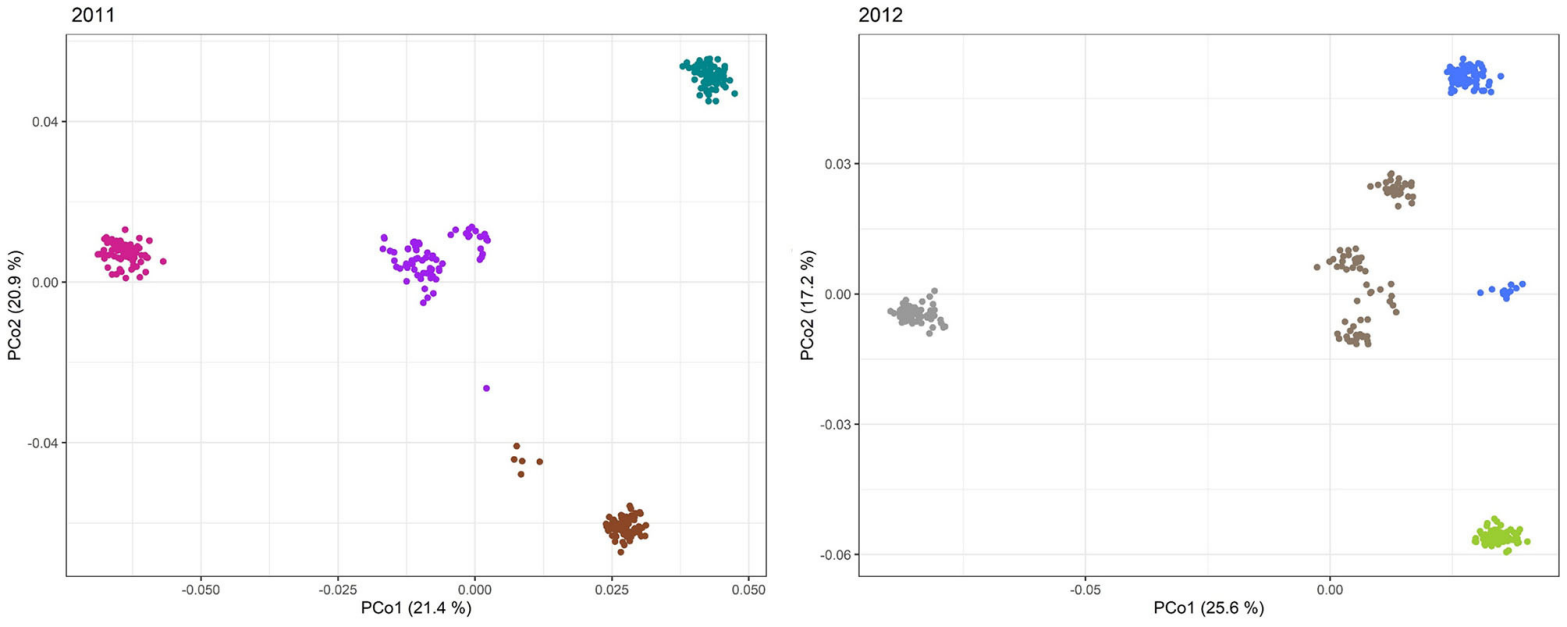
Principal coordinates analysis (PCoA) of the 298 and 305 single cross rye hybrids in 2011 and 2012. Scatter plots of the first two principal coordinates based on genotyping data of 593 and 663 SilicoDArT™ markers, respectively. The horizontal and vertical coordinates represent PCo1 and PCo2. Each dot represents an accession. The samples are clearly stratified by ancestry. Samples are colored according to group assignment of the structure analysis.

**Figure 7:**
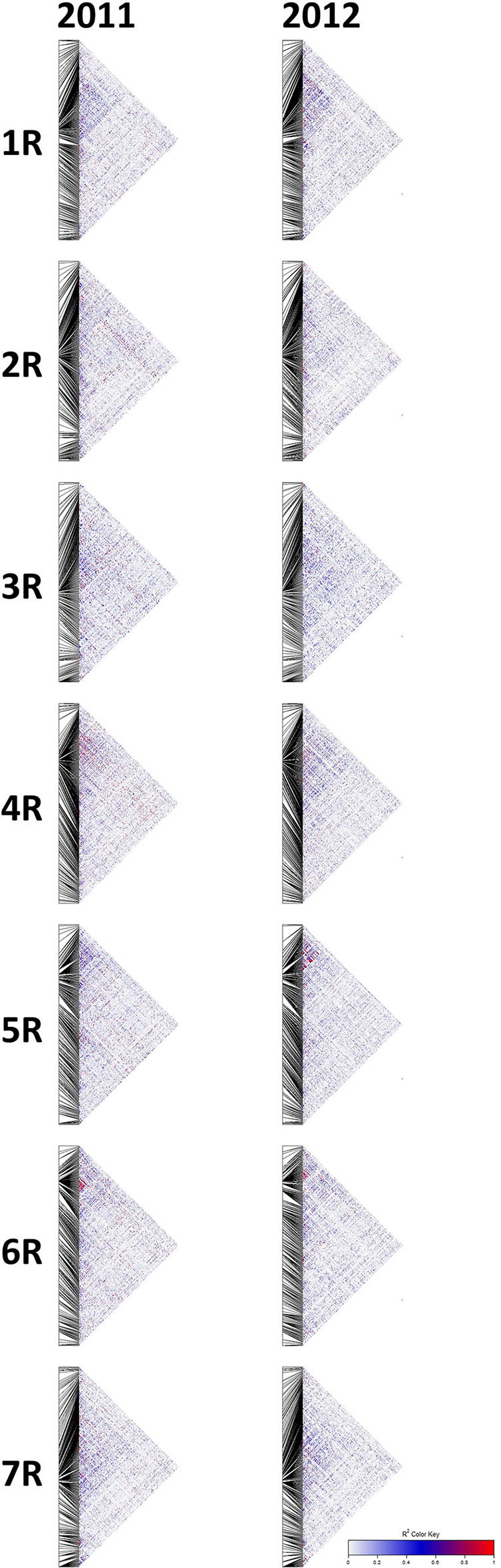
Heatmaps of squared allele frequency correlation values (${\text{r}}_{\text{vs}}^{2}$) corrected by relatedness of genotyped individuals and the structure of the sample to display intra-chromosomal linkage disequilibrium (LD) for both germplasm collections. ${\text{r}}_{\text{vs}}^{2}$ in experimental hybrid rye genotypes based on 2,563 and 2,790 polymorphic SNP markers identified by DArTseq™. The color legend for ${\text{r}}_{\text{vs}}^{2}$ values is given on the right side.

Based on Evanno's DeltaK method, we determined k = 4 as the most probable number of groups in each of the two sets of experimental hybrids (Figure 5). The principal coordinates analysis confirmed this major population substructure and revealed that the first two principal coordinates explained 42.3 and 42.8% of the genetic variation within the 298 and 305 rye hybrids (Figure 6). The grouping of experimental hybrids into subpopulations referred mainly to the individual pollen parent genotypes. We have integrated 13,337 SilicoDArT™ and 3,711 novel SNP markers in the genetic linkage map for the RIL-population L2039-N x DH. The advanced map covered 1946.4 cM of the rye genome with map length of the seven rye chromosomes varying between 227 cM of chromosome 3R and 340 cM of chromosome 4R (Supplementary Table 2). The 3,711 novel SNP markers were equally distributed across the genome with SNP numbers varying between 402 on chromosome 3R to 577 on chromosome 4R (Supplementary Table 2). We have used this high-density map to investigate the LD in the analyzed experimental hybrids. Visual inspection of intra-chromosomal heatmaps from all segregating markers depicts no noticeable differences in the LD structure between both germplasm collections (Figure 7). In the distal region of chromosome 6RS, a small distinct LD block was observed in both populations. Mean ${\text{r}}_{\text{vs}}^{2}$ of individual chromosomes ranged from 0.07 for 1R in 2011 to 0.087 for 4R in 2011 (Table 4). In the collection of 298 single cross hybrids 2011, the critical value of ${\text{r}}_{\text{vs}}^{2}$ (basal LD) is ${\text{r}}_{\text{vs}}^{2}$ = 0.2467. Similarly, in the cohort of 305 single cross hybrids 2012, the critical value is ${\text{r}}_{\text{vs}}^{2}$ = 0.2208. Based on these critical values, the intrachromosomal LD decayed between 1.51 (2R in 2011) and 4.6 cM (6R in 2012) for the individual chromosomes in the germplasm collection (Table 4). The mean LD decay over the whole genome was calculated as 2.2 cM (2011) and 2.77 cM (2012).

**Table 4:** Estimates of LD as well as distances at which LD decays below background LD for single cross hybrids in 2011 and 2012.

**Year**	**2011**		**2012**	
**Chromosome**	**Mean ${\text{r}}_{\text{vs}}^{2}$**	**LD decay**	**Mean ${\text{r}}_{\text{vs}}^{2}$**	**LD decay**
1R	0.070	1.75	0.082	3.55
2R	0.077	1.51	0.072	1.81
3R	0.079	1.7	0.078	2.01
4R	0.087	2.17	0.08	2.52
5R	0.079	3.34	0.079	4.3
6R	0.081	3.35	0.078	4.6
7R	0.074	1.71	0.077	2.45
Whole genome	0.078	2.2	0.078	2.77

### GWAS of Agronomic and Quality Traits

**Figure 8:**
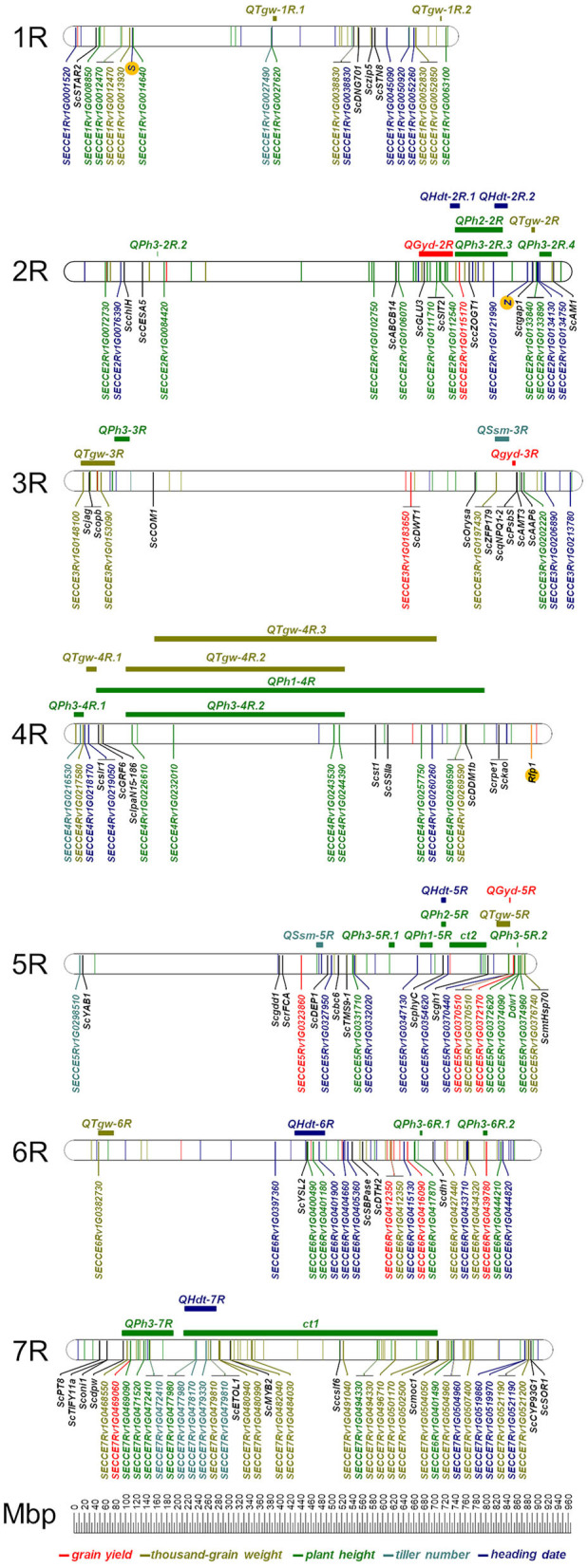
Physical position of cross-validated SNPs in protein coding sequences of the ‘Lo7' genome assembly detected in the GWAS for grain yield, thousand-grain weight, plant height, tiller number and heading date. The positions of both self-incompatibility loci, *S* and *Z*, the restorer-of-fertility locus *Rfp1* depicting the rye's unique reproduction biology, and the GA-sensitive dwarfing gene *Ddw1* are given as well. For ‘Lo7' orthologs of cloned rice QTL the corresponding rice gene symbols were adapted to rye. The positions of the markers in the ‘Lo7' physical map are given in Mbp. The horizontal bars and QTL symbols indicate the position of grain yield (QGyd-2R), heading date (QHdt), thousand-grain weight (QTgw), plant height (QPh), and spikes per squaremeter (QSsm).

**Figure 9:**
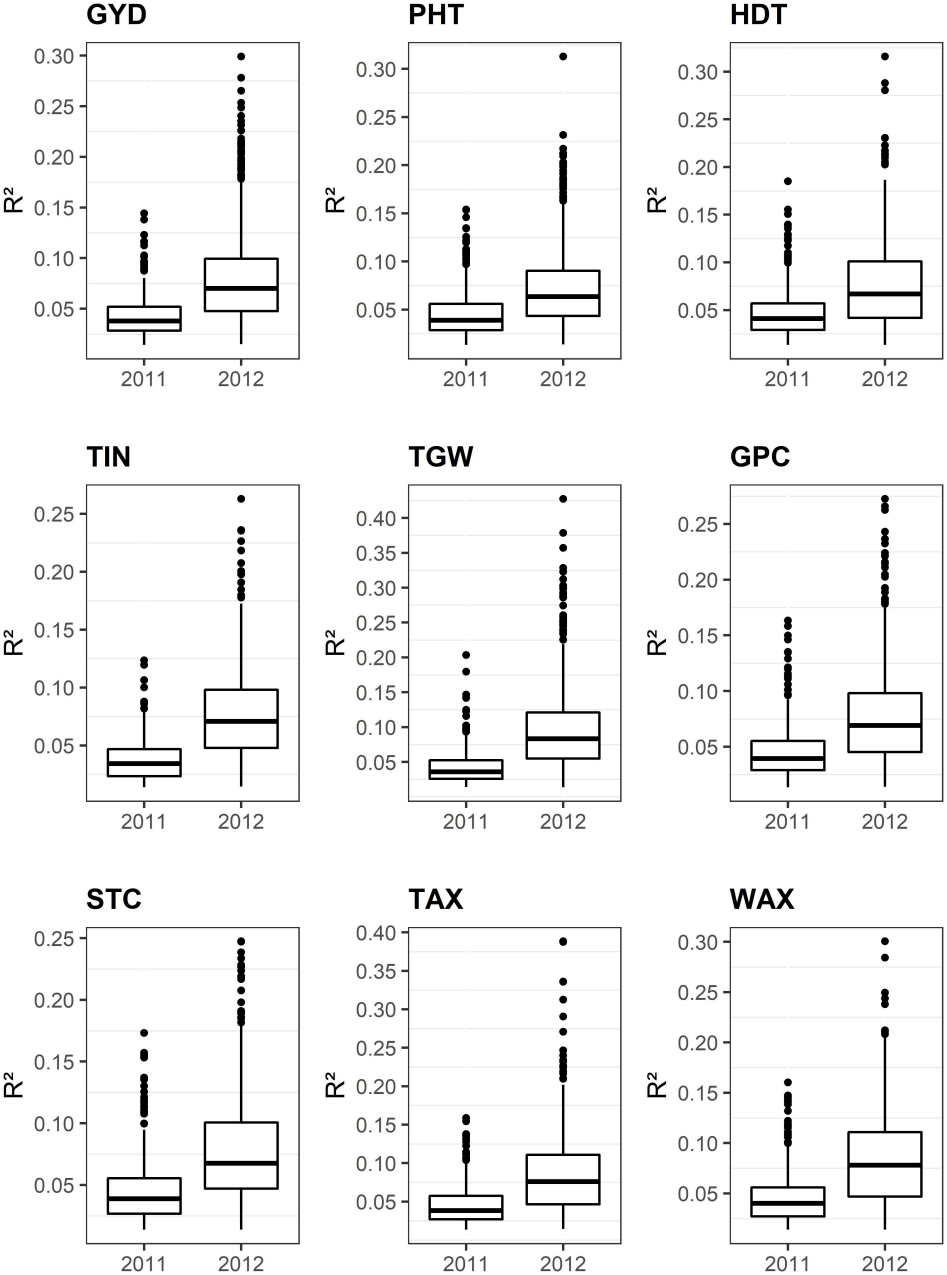
Box plots illustrating the phenotypic variances (R^2^) explained by individual associated SNPs. GYD, grain yield; TGW, thousand-grain weight; PHT, plant height; HDT, heading date; TIN, tiller number; GPC, grain protein content; STC, starch content; TAX, total arabinoxylan content; WAX, water extractable arabinoxylan content.

Out of 20,232 DArTseq™-derived SNP tags, 228 (1.1%) are covered by the Rye 600k genotyping array (Supplementary Table 3) and 10,712 (52.9%) mapped to the ‘Lo7' pseudomolecules (Supplementary Table 2). Map positions could be determined for 10,171 (94.9%) of these SNP tags with 1,302 located on chromosome 1R, 1,534 on 2R, 1,388 on 3R, 1,498 on 4R, 1,474 on 5R, 1,464 on 6R, and 1,520 on 7R (Supplementary Table 2). About 3,901 SNPs (36.4%) represent predicted coding sequences in the ‘Lo7' genome assembly. Comparison between 2,149 (21.1%) genetically mapped SNPs revealed almost perfect collinearity to the physical map. GWAS revealed that between 25 of these SNPs on chromosome 1R and 278 on chromosome 6R were significantly (*p*_adj_ <0.05) associated with PHT, HDT, and the studied agronomic traits (Supplementary Table 4). We identified between 34 on chromosome 1R and 186 on chromosome 7R significantly (*p*_adj_ <0.05) associated SNPs for quality traits (Supplementary Table 4). For GYD, 38 SNP loci showed significant association (*p*_adj_ <0.05) in both years. For all remaining traits, this value ranged between 15 and 132 significant SNPs (Table 5, Figure 8, and Supplementary Table 4). The explained phenotypic variance (R^2^) of the individual QTL ranged from 1.4 to 29.9% for GYD, 1.3 to 31.3% for PHT, 1.4 to 31.6% for HDT, 1.4 to 26.3% for TIN, 1.4 to 42.7% for TGW, 1.4 to 27.3% for GPC, 1.4 to 24.7% for STC, 1.3 to 38.8% for TAX, and 1.4 to 30.1% for WAX (Figure 9, Supplementary Table 4). In total, between 100 and 566 of the associated SNPs represent predicted protein-coding genes of the ‘Lo7' assembly (Supplementary Table 4). Between 9 and 35 marker-trait associations (MTA) in protein-coding sequences could be cross-validated in both datasets (Table 5, Figure 8). Nine of the cross-validated MTA for PHT are located within the *QPh3-7R* interval, including SECCE7Rv1G0469090, SECCE7Rv1G0471520, and SECCE7Rv1G0472410, respectively. SECCE6Rv1G0401900 ranks among two MTA for HDT located within *QHdt-6R*. Among the MTA for TGW, we identified the polyubiquitin-encoding gene SECCE5Rv1G0370510 residing within *QTgw-5R*. SECCE6Rv1G0382730, encoding an Embryo-defective protein, mapped to *QTgw-6R*. We illustrate the identification of putative causal genes associated with variation in target traits of rye breeding using plant height, thousand-grain weight, and yield, as well as arabinoxylan content as examples.

**Table 5:** Survey on cross-validated marker/trait-associations for agronomic traits, plant height, heading date and grain quality traits mapped in the ‘Lo7' genome assembly.

**Lo7**	**PHT**	**HDT**	**GYD**	**TIN**	**TGW**	**GPC**	**STC**	**TAX**	**WAX**
0R	3	7	10	3	7	4	4	4	6
1R	13	16	1	1	17	2	6	19	7
2R	28	13	3	1	11	6	15	8	4
3R	11	9	5	1	14	1	10	18	7
4R	14	8	3	2	5	2	1	8	11
5R	16	14	4	1	7	6	8	15	3
6R	11	31	11	0	18	9	6	2	9
7R	20	9	1	6	53	15	37	12	3
Σ	116	107	38	15	132	45	87	86	50
cds	35	31	13	9	32	13	27	31	15

*GYD, grain yield; PHT, plant height; HDT, heading date; TIN, tiller number; TGW, thousand-grain weight; GPC, grain protein content; STC, starch content; TAX, total arabinoxylan content; WAX, water-extractable arabinoxylan content. 0R: unknown pseudomolecule; cds: protein coding sequence*.

### Candidate Genes for Plant Height

We observed 483 SNPs associated with PHT in ‘Lo7' protein-coding genes (Supplementary Table 4). Fifteen (3.1%) of these genes are orthologs of cloned rice QTL, including the Gibberellin-insensitive dwarfing gene *SLR1*, the epigenetic regulator *Decrease in DNA Methylation 1* (*DDM1*), the lectin receptor-like kinase *SIT2*, the phosphate transporter *PT8*, and the transmembrane ABC transporter *STAR2* (Supplementary Table 5). *SIT2* turned out to be associated in both years. Altogether, 35 SNPs in ‘Lo7' gene models were associated in both years (Table 5). The SNP in SECCE1Rv1G0063100 encoding a putative translation initiation factor IF-2 explains more than 10% of the phenotypic variance in both years.

### Candidate Genes for Thousand-Grain Weight

**Figure 10:**
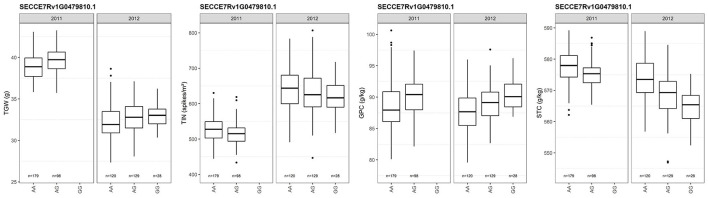
Box plots depicting the effect of SNP genotypes of the *ETHYLENE OVERPRODUCER 1-like* gene SECCE7Rv1G0479810 on TGW, TIN, GPC and STC in rye. Dots represent outliers. TGW, thousand-grain weight; TIN, tiller number; GPC, grain protein content; STC, starch content.

We detected 685 SNPs associated with TGW in ‘Lo7' protein-coding genes (Supplementary Table 4). Fourteen (2%) of the ‘Lo7' protein-coding genes are orthologs of cloned rice QTL, including the zinc finger protein gene *ZFP179*, a cytochrome P450 protein-encoding gene, and an *ETHYLENE OVERPRODUCER 1*-*like* gene (*OsETOL1*) (Supplementary Table 5). The *OsETOL1* ortholog of SECCE7Rv1G0479810 affects TIN, GPC, and STC in rye as well. Contrasting effects on TIN and STC as compared with TGW and GPC could be observed for the A547G SNP in SECCE7Rv1G0479810 (Figure 10). The experimental hybrids evaluated in 2012 represented three SNP genotypes, while, in 2011, only two genotype classes could be detected. Major effects (R^2^ > 10%) on TGW could be observed for 9 of the 525 SNPs (1.7%) associated in 2011, including the predicted sucrose transporter SECCE7Rv1G0456720 and for 535 of the 1,465 (36.5%) SNPs associated in 2012 (Supplementary Table 4). The cross-validated effects of 132 SNPs on thousand-grain weight explained between 1.4 and 30.3% of the phenotypic variance. The protein-coding sequences represented by these SNPs include SECCE1Rv1G0038830, a nitrate/peptide transporter; SECCE1Rv1G0052830, encoding a methyltransferase, the invertase inhibitor SECCE3Rv1G0153090; SECCE5Rv1G0376740, encoding a basic helix-loop-helix (bHLH) DNA-binding superfamily protein; SECCE6Rv1G0427440, encoding a jasmonate O-methyltransferase; and SECCE7Rv1G0479810, encoding an ethylene-overproduction protein.

### Candidate Genes for Grain Yield

We identified 19 (3.8%) out of 500 SNPs associated with GYD in protein-coding genes of the ‘Lo7' assembly as orthologs of cloned rice QTL (Supplementary Table 5). These included the WUSCHEL—related homeobox transcription factor *DWT1*, the Zinc-finger transcription factor *YABBY1*, the gene *DTH2* promoting heading under long-day conditions, the cellulose synthase *bc6*, the soluble starch synthase IIa, the Cytochrome P450*CYP96B4*, the metal-nicotianamine transporter *YSL2*, the amino acid transporter AAP6, the ATP-binding cassette protein, encoding gene *ABCB14*, the putative potassium efflux antiporter *Albino midrib 1* (*AM1*), the Zinc finger family protein encoding gene *sor1*, and the transmembrane ABC transporter *STAR2*. While the SNP representing the *DWT1*-ortholog in rye was associated in both years, the SNPs in the rye orthologs of *YABBY1* and *ABCB14* were associated in 2011 and the remaining SNPs in 2012. Among the 337 SNPs associated in 2011, eight (2.4%) explained more than 10% of the phenotypic variance. Three of these SNPs represent genes encoding a cytochrome P450 protein, a putative late embryogenesis abundant (LEA) hydroxyproline-rich glycoprotein, as well as an ARM repeat superfamily protein. Likewise, 101 (20.2%) among the 500 SNPs in protein-coding sequences associated in 2012 had major effects (R^2^ > 10%) on GYD. Except for 6, the 38 SNPs associated in both years had minor effects (R^2^ < 10%) on grain yield. The genes represented by these major effect SNPs include SECCE3Rv1G0183650 encoding, a WUSCHEL homeobox protein, SECCE5Rv1G0323860.1, a HAESA-like LRR receptor protein kinase, SECCE6Rv1G0412350, a flavonol synthase, SECCE6Rv1G0416090, a putative Snf1-related kinase interactor, SECCE7Rv1G0469060, a Gibberellin 2-beta-dioxygenase, and SECCEUnv1G0531960, a BEL1-like homeobox protein.

### Candidate Genes for Arabinoxylan Content

We discovered 416 and 433 SNPs associated with TAX and WAX, respectively, in ‘Lo7' protein-coding genes (Supplementary Table 4). Eleven (2.6%) and nine (2.1%) of the ‘Lo7' protein-coding genes are orthologs of cloned rice QTL, including the cZ-O-glucosyltransferase *cZOGT1*, an embryo and endosperm development-involved cyclin and the transmembrane ABC transporter *STAR2* (Supplementary Table 5). Major effects (R^2^ > 10%) on TAX and WAX in 2011 were observed for 17 and 23 SNPs, respectively. Corresponding protein-coding sequences represented, among others, the receptor protein kinases SECCE2Rv1G0135820 and SECCE4Rv1G0223400, and the hexosyltransferase SECCE5Rv1G0328330. In 2012, 249 and 202 SNPs revealed major effects (R^2^ > 10%) on TAX and WAX. These SNPs described, among others, the α-glucosidase SECCE3Rv1G0149680 and the xyloglucan α-1,6-xylosyltransferase SECCE6Rv1G0407620. Major effects (R^2^ > 10%) on TAX were observed for SNPs in the Glycosyltransferase-encoding genes SECCE3Rv1G0197970 and SECCE7Rv1G0456850, and minor effects (R^2^ < 10%) on TAX and WAX for SNPs in six further Glycosyltransferase-encoding genes (Supplementary Table 4). The predicted glycosyltransferase SECCE3Rv1G0150570 revealed a significant association with TAX and WAX content. Across eight environments, we detected 31 and 15 cross-validated SNPs in protein-coding genes associated with TAX and WAX, respectively (Table 5, Supplementary Table 4).

### Co-Localization of QTL

We identified 34 cross-validated SNPs in 33 protein-coding sequences associated with more than one trait (Supplementary Table 6). The number of cross-validated MTA ranged from four (GYD) to 16 (TGW). On average, each of the seven ‘Lo7' pseudomolecules carry 4.6 of these gene models, 35.3% of the predicted rye genes mapped to chromosome 7R. Three protein-coding sequences were associated with four traits: next to the already described associations for SECCE7Rv1G0479810 (Figure 10), SECCE5Rv1G0347130, encoding a putative transmembrane protein, was associated with HDT, STC, TAX, and WAX, and SECCE7Rv1G0477980, encoding a cysteine-rich receptor-kinase-like protein, with PHT, TIN, STC, and TAX. Likewise, SECCE1Rv1G0052830 and SECCE7Rv1G0484030, encoding a methyltransferase and a cytochrome P450 family protein, were associated with TGW, GPC, and STC, while the predicted chitinase-encoding gene SECCE7Rv1G0521190 was associated with TGW, HDT, and WAX.

## Discussion

To fully exploit the recently published reference genome sequence data for rye (Li G. et al., 2021; Rabanus-Wallace et al., 2021), a systematic approach for the discovery of gene function is required. Genome-wide association mapping was hailed as the key gene discovery paradigm to translate the expectations connected to high-quality genome sequences into practice, providing the insights needed to develop better diagnostic, prognostic, and preventive strategies for different traits (Liu and Yan, 2019; Loos, 2020). Indeed, GWAS has become a central approach to studying the natural variation and mapping quantitative traits of cereals (Alqudah et al., 2019). However, in contrast to wheat and barley, target genotypes in outbreeding rye are highly heterozygous. This results in decreasing genotypic correlation between the line *per se* and test cross performance with the increasing complexity of a trait (Sprague and Tatum, 1942; Miedaner et al., 2014). We have, thus, studied two large multiple-hybrid populations to dissect the genetic architecture of important agronomic and quality traits in rye.

### Prospects and Limits of Field Phenotyping in Rye

The rich genetic diversity in random mating rye populations has been depicted using DNA marker technologies (Hagenblad et al., 2016; Schreiber et al., 2018; Sidhu et al., 2019; Hawliczek et al., 2020). In the present study, we observed significant genotype variation (*p* < 0.05) for major target traits in a rye improvement program. The observed significant correlations reveal that complex target traits of rye breeding do not evolve independently. However, as weak and moderate correlations between traits dominate, the substitution of an inexpensive measurement for a correlated expensive phenotype in variety development is no constructive option. The strong to moderate negative correlation (r = −0.9 and r = −0.39) between GPC and STC in both data sets is well-known from studies in wheat (Muqaddasi et al., 2020; Yang et al., 2020). In contrast to previous research in biparental populations (Miedaner et al., 2012), the observed correlation between GPC and STC could be explained by the co-localization of QTL in our populations. The intermediate to high trait heritabilities (H^2^) for GYD, PHT, and TGW compare well with previously published heritability estimates in biparental (Miedaner et al., 2012, 2018; Hackauf et al., 2017a) and multiple-hybrid populations (Auinger et al., 2016) of rye. This applies to STC (Miedaner et al., 2012) and HDT (Hackauf et al., 2017a) as well. The high heritability estimates for GPC, TAX, and WAX refer to high genetic variance and low error variance that is sufficiently powerful to detect additive gene variants even with minor effects. Heritability estimates of TIN compare well with data from wheat (Bilgrami et al., 2020) and uncovered a phenotyping bottleneck in our study. As we have studied TIN in both years in a large biparental population as well (Hackauf et al., 2017a) and due to a low throughput in assessing this trait (Wu et al., 2019; Reynolds et al., 2020), we were able to phenotype TIN in three environments only. At this point, the present study emphasizes that automated, nondestructive methods of phenotyping tiller traits at a high spatial resolution and high throughput for large-scale assessment of small grain cereal accessions are urgently needed (Wu et al., 2019). The relevance of this conclusion is supported by previous research that identified genetic gains achieved in ear density, i.e., the number of effective tillers, as the main factor responsible for the progress in grain yield of hybrid rye cultivars (Laidig et al., 2017).

### Novel Insights in Rye's Adaptation to Drought Stress

The atypical extreme climatic conditions in 2018 demonstrated that environmental impact is becoming increasingly relevant to agricultural production in Europe (Beillouin et al., 2020). Europe accounts for around 20% of the global cereal production (Schils et al., 2018) and is the main rye-producing area globally (FAOSTAT, 2020). Among the small grain winter cereals produced in Europe, rye revealed the lowest yield reduction compared to wheat, barley, and triticale when rainfall was fully excluded by means of rain-out shelters from tillering until harvest (Schittenhelm et al., 2014). The main driver enabling the performance of rye on light soils with low fertility and low water capacity is its highly developed root system. The entire root system of a single rye plant consisted of 13,815,672 branches, with a total length of 622 km, a surface area of 401 m^2^, and a total root hair length of 11,000 km (Dittmer, 1937), which facilitates a very efficient uptake of water and nutrients (Paponov et al., 1999; Yeo et al., 2014). Irrespective of the powerful root system, average drought-induced grain yield reduction of 23.8% has been reported for hybrid rye in non-irrigated compared with the irrigated regime under natural drought stress conditions (Hübner et al., 2013), while up to 57% grain yield reduction was observed in controlled environments under different drought regimes (Kottmann et al., 2016). Notably, knowledge of physiological mechanisms of drought tolerance in rye is scarce (Hübner et al., 2013). As demonstrated on a large scale in the present study, natural drought stress conditions in Europe were detrimental in 2011 to grain yield of rye as compared with less-osmotic stress conditions in 2012. Our comprehensive phenotyping identified morphological adjustment of plant height and tiller number as drought stress responses of rye. Supported by a significant (*p* < 0.001) negative correlation between PHT and STC, the higher sink activity of grains under drought suggests a mobilization and reallocation of stem reserves to the grain, as has been observed in Sorghum (Blum et al., 1997). Interestingly, neither STC nor GPC was correlated with TGW under drought.

The significant opposite correlations between GYD and STC as well as PHT and STC in 2011 demonstrate that the large stem reserve storage in modern hybrid cultivars represents an adaptation strategy of rye for stable grain filling under osmotic stress. This protective measure needs to be considered when using the gibberellin (GA)-sensitive dwarfing gene *Ddw1* (Börner and Melz, 1988) as a breeder's option to improve plant height and lodging resistance in hybrid rye (Braun et al., 2019) and asks to search for an optimum in plant height of semi-dwarf rye hybrids. However, the significant negative correlations between PHT and STC in both years distinguish the potential of further improvements in PHT as a viable option to tap currently unused potential for optimized dry matter allocation to the rye grain. Indeed, as no correlation between GYD and PHT was found in both years, this approach is not compromised by a relationship between grain yield and tall plant stature, as has been reported in a biparental population in rye (Miedaner et al., 2012). Significant (*p* < 0.01) positive correlations with PHT in both years indicate that HDT might be involved as a confounding variable. Although early heading date and a shorter vegetative phase were significantly (*p* < 0.001) correlated with grain yield under less-osmotic stress conditions in 2012, drought escape through earlier heading could not be identified as an adaptive mechanism of rye in 2011, as indicated by a correlation coefficient between GYD and HDT of almost zero. Further research directed to the collection of drought tolerance traits that are not easy to measure under field conditions, and the assessment of rye using state-of-the-art phenotyping in controlled environments (Langstroff et al., 2021) is necessary to consolidate the relevance of our conclusion. The substantially increased grain arabinoxylan content in osmotically stressed rye is a striking phenotype that will subsequently be discussed.

### High Levels of Genetic Diversity in Elite Rye Germplasm

The accessibility of distinct heterotic gene pools is the central pillar for the breeding of hybrid cultivars (Labroo et al., 2021). We investigated the extent of genetic differentiation, population clustering, and patterns of relationship among a diverse set of elite rye inbred lines based on SNP markers. All multivariate methods revealed the presence of two major groups, which was in perfect agreement with pedigree information, as all lines with similar pedigree clustered into the same group. The observed differences between inbred lines from the ‘Petkus' and ‘Carsten' pool concerning the average heterozygosity reflected that line development in the ‘Carsten' pool is currently not going for complete homozygosity since a genetically broader synthetic composed of two less inbred lines is a more secure pollinator due to a longer pollen-shedding period (Geiger and Miedaner, 2009). We observed a high pairwise differentiation (*F*_*ST*_) among the ‘Petkus' and ‘Carsten' populations, which coincides with the identified heterotic pattern in rye (Hepting, 1978) and indicates that these groups obviously followed different domestication and/or artificial selection paths. Our results generally agree with previous studies (Bauer et al., 2017; Vendelbo et al., 2020) that reported high genetic differentiation between parental populations of two other hybrid rye breeding programs as well. It is worth noting that the *F*_*ST*_ between elite ‘Petkus' and ‘Carsten' rye inbred lines estimated in the present study is higher than the differentiation (*F*_*ST*_ = 0.10) estimated from single nucleotide polymorphisms between elite and exotic wheat lines (Boeven et al., 2020). As the genetic diversity described in the analyzed rye germplasm has successfully been used to exploit commercial heterosis (Bundessortenamt, 2013), data presented in the present study may illustrate the (i.) sought genetic diversity and (ii.) command variable for marker-assisted management of elite germplasm pools in wheat hybrid breeding programs. Although comprehensive analyses have recently reported the genetic distance between parental populations as a crucial parameter to maximize the exploitation of heterosis for rye (Vendelbo et al., 2020) and wheat improvement (Boeven et al., 2020), it needs to be considered that heterotic response between genetically divergent groups cannot be predicted from genetic distances based on DNA markers but must be evaluated in field trials (Melchinger, 1999). Irrespective of that, SNP markers with high polymorphism information content (PIC) and Nei's gene diversity across ‘Lo7' pseudomolecules may be useful to develop a subset of SNPs for routine studies where only a small to moderate number of SNPs are needed, as is the case in mapping, marker-assisted recurrent selection, marker-assisted backcrossing, and quality control applications in rye.

### Rapid Decay of LD in Elite Rye Germplasm

Almost 99% of the SNP tags described in the present study are not represented by the Rye 600k SNP array (Bauer et al., 2017). The novel high-density map further extends previous versions established for the L2039-N x DH mapping population based on gene-based SNPs (Martis et al., 2013), as well as first-generation DArT™ markers (Hackauf et al., 2017a). The markers are evenly distributed through the ‘Lo7' genome sequence, and the length of the linkage map is in the same order as other genetic maps in rye (Martis et al., 2013; Milczarski et al., 2016; Borzecka et al., 2018).

We observed low levels of LD extend across the entire genome in elite rye germplasm and in a similar order as most recently estimated for wheat (Liu et al., 2020). A rapid decline of LD fits to the expectation for an outcrossing species with low ancestral LD (Auinger et al., 2016). More recently, a higher mean genome-wide LD with several distinct blocks of strong LD has been observed in another rye germplasm (Vendelbo et al., 2020). This unexpected attribute refers to the use of the Gülzow (G) male-sterility system (Melz et al., 2003) as a hybridization system that obviously constitutes a population-determining parameter. Indeed, the G cytoplasm belongs to a plasmotype that is common in the Central European rye gene pool (Stojałowski et al., 2008). The spread in random-mating populations of cereal rye indicates that this plasmotype obviously provides a fitness advantage in female function [see Rieseberg and Blackman (2010) for review] that, in turn, resulted in a high frequency of restorer alleles (Łapiński and Stojałowski, 2003). This has a strong impact on practical applications. The unusual high LD reported by Vendelbo et al. (2020) demonstrates that, due to a low frequency of reliable maintainer genotypes, the development of male sterile seed parent lines for breeding of G-type CMS hybrid rye is a challenging task. In contrast, the CMS-inducing P cytoplasm used in the present study is easy to maintain and enables an unbiased capture and management of genetic diversity in the seed parent pool. The rapid rate of LD decay in P-type CMS hybrids promises a high resolution in GWAS and suggests to advance research on rye as a complement to barley, the model for the genetics and genomics of the Triticeae tribe (Han et al., 2020; Jayakodi et al., 2020) that features a comparatively higher mean LD (Malysheva-Otto et al., 2004; Comadran et al., 2009; Rode et al., 2012; Bellucci et al., 2017). As LD has been reported to decay rapidly within ~520 bp on average in rye (Li et al., 2011), follow-up experiments based on a higher SNP density than in the present study will enable to accurately predict LD beyond genetic maps.

### Bridging the Genotype-Phenotype Gap in Rye

We integrated the novel SNP markers in the L2039-N x DH genetic map and the recently established rye reference genome. We obtained both genetic and physical positions for markers representing genetic diversity that is managed in the elite germplasm of a hybrid rye breeding program. The DArT™ genetic map enabled the confident chromosomal localization of 90 SNP markers of unknown physical position in the ‘Lo7' genome assembly to genome-specific regions and provides map position of 23 protein-coding genes. Both, the ‘Lo7' reference genome sequence as well as the high-density map as a complement, will support marker development, marker-assisted selection (MAS), gene discovery and isolation, and, as demonstrated in the present study, GWAS. Furthermore, functional markers can explain a large part of the genetic variance, which may improve the predictive ability of genomic selection models in plant breeding programs (Liu et al., 2019). We ascertained GWA QTL that overlapped with previously identified QTL for plant height and agronomic traits, including grain yield (Hackauf et al., 2017a; Miedaner et al., 2018), by integrating the latter into the ‘Lo7' assembly. Except for QTL residing on chromosome 4R, the physical distance covered by the QTL is low. It is currently unclear whether the QTL on chromosome 4R resides in a rarely recombining region, as has been demonstrated for the GA-insensitive dwarfing gene *compactum1* (*ct1*) mapping to a region with a high ratio of physical to a genetic distance of 122.4 Mbp/cM on chromosome 7R (Hackauf et al., 2021). Alternatively, the observation may owe to the many inversions and large structural rearrangements that have been observed among non-‘Lo7' Secale genotypes relative to ‘Lo7' (Rabanus-Wallace et al., 2021). Likewise, the large 4R intervals may be attributed to copy number variation for the QTL markers in the biparental mapping population that is not mirrored in the ‘Lo7' genome assembly.

Anchoring the SNPs to the ‘Lo7' reference genome is in the same order of magnitude as recently reported for the alignment of SNPs and SilicoDArT™ markers to the hexaploid wheat RefSeq v1.0 reference genome (Sansaloni et al., 2020). The ‘Weining' genome assembly (Li G. et al., 2021) offers a further resource to anchor additional rye DArTseq™ SNP tags with locations in physical space. Alignment of (i) DArTseq™ SNP tags representing protein-coding genes, as well as (ii) the corresponding protein-coding genes in rye and wheat, will support the identification of diversity that is associated with important agronomic and quality traits but that may be missing in current wheat-improvement programs. As expected, 60–70% of the SNP markers were in intergenic regions, reflecting that genic regions are evolutionarily more conserved compared with intergenic regions, which evolve faster and accumulate higher levels of polymorphism. However, part of these SNPs may reside in promoters and regulatory elements and may represent functional markers as well.

The random-mating populations ‘Halo' and ‘Carokurz' as well as the two inbred lines ‘Lo7' and ‘Lo225,' represent the heterotic gene pools ‘Petkus' and ‘Carsten,' respectively, and revealed significant differences in monoploid genome size (Rabanus-Wallace et al., 2021). This striking observation suggests the occurrence of structural variants (SVs) in the rye genome. Indeed, Hi-C asymmetry plots revealed SVs between the genomes of ‘Lo7' and representatives of the genus Secale, including the inbred line ‘Lo225' (Rabanus-Wallace et al., 2021). In the present study, DArTseq™ detected more than 27k SVs in the analyzed rye germplasm markers that could be mapped to the ‘Lo7' genome assembly (data not shown). The impact of these SilicoDArT™ on rye phenotypes deserves further research.

### Controlling the Rate of False Discoveries

Control of the false discovery rate (FDR) in genetic association studies has become an increasingly appealing and accepted target for multiple-comparison adjustment (Brzyski et al., 2017). In order to control the false positive rate in multiple testing procedures, we have adjusted the level of statistical significance (p-value) of a single test so that the overall false control is still at a low level. While correction methods based on the family-wise error rate provide the most stringent control of false positives, we applied the FDR standard method according to Benjamini and Hochberg (1995) to better balance the false and hit and increase the power of GWAS (Zheng and Zhuo, 2017), as recently adopted in wheat (Ladejobi et al., 2019). In addition, we counterbalanced potential false positive associations due to statistical inferences (Liu and Yan, 2019) and other unaccounted factors, such as low-accuracy genotype calling at some loci (Browning and Yu, 2009) or small population size (Finno et al., 2014) by cross-population and cross-species validation. The successful validation of candidate genes in the two different sets of experimental hybrids renders true associations more likely. This approach enabled to achieve high power in our rye GWAS to detect associations between SNPs and traits. The identified marker-trait associations reflect an appropriate sample size and substantial genetic diversity that determined the phenotypic variance of target traits in the studied elite germplasm. The rapid LD decay and rich genetic diversity of outbreeding species like rye are known to increase power in GWAS as compared with self-fertilizing species (Huang and Han, 2014). The GWAS reported in the present study serves as a foundation experiment by providing insights into the genetic architecture of important traits for rye improvement, allowing the targeted choice of parents for subsequent experiments, like candidate gene knock-out, over-expression, or genetic complementation, that are, in any case, indispensable to validate genes underlying the analyzed traits. Hybrid rye breeding offers a further and pragmatic cross-validation strategy of a single genomic locus with possible influence on the phenotype. The high-quality genome sequences of rye (Li G. et al., 2021; Rabanus-Wallace et al., 2021) enable the extension of sequence information for an efficient transformation of DArTseq™-based SNPs to single-plex Kompetitive Allele-Specific PCR (KASP) (Semagn et al., 2014) assays. These KASP markers will be used in progenies segregating for the superior (S) or inferior (I) SNP alleles to select and establish near-isogenic genotype bulks (NIB) of homozygous F3 lines, which will serve as pollinators in crosses with male-sterile single-cross testers between isolation walls (Supplementary Figure 1). The genetic makeup of these hybrids enables to precisely estimate phenotypic effects, recorded in target environments of rye production, as the difference (ΔS-I) between the means of individual NIB partners, which either carry the S or the I allele at the candidate gene. This approach can, henceforth, take on central importance in ongoing efforts to isolate and characterize specific loci and bridge the genotype-phenotype gap for precision breeding in rye. However, it needs to be considered that the correlation between variants at a locus due to LD in experimental hybrids that have been established based on elite inbred lines further challenges the identification of causal variants, just as it is hard to find true signal overlaps between a GWAS and a QTL signal. Approaching target genes in gametes captured from random-mating rye populations with rapid decay of LD (Li et al., 2011) offers to overcome this challenge as well.

To conclude, the SNP catalog published with this paper can assist scientists to discover and use functional diversity in rye and related Triticeae species that may be essential for meeting the compulsions to act in modern agriculture under the directive of the multiple challenges concerning global food security (Lal, 2016; Liu et al., 2020).

### An Extended View on the Genetic Basis of Variation for Complex Traits in Rye

Knowledge of genomic regions controlling complex traits is the key to our understanding of mechanisms behind trait architecture and for using them in marker-assisted crop improvement programs. In the present study, both progeny sets of experimental rye hybrids from controlled crosses resulted in adequate statistical power to detect QTL, including those with small effects, and precisely map them in the ‘Lo7' genome assembly. For all traits studied, a substantial proportion of the phenotypic variation can be explained with few loci of large effect, with the remainder due to numerous loci with small effect. QTL with large effects accounting for substantial proportions of phenotypic variance is well-known (Remington and Purugganan, 2003; Mackay, 2004; Roff, 2007) and has been identified in biparental rye mapping populations as well (Miedaner et al., 2012; Hackauf et al., 2017a). The large-effect QTL observed in the present study fit the model developed by Orr (1998), which suggests that natural selection validates mutations with large effects at the beginning of an adaptation process with a maximum of adaptive space, while, later on, in the process when the organism has essentially reached its optimum state, the space is narrowed and successful mutations must have smaller effects. In rye, an impressive selection gain for TGW from initially 19 g up to 57 g in a random mating population within two decades of breeding (Dill, 1983, 1989) supports the assumption that major genes controlling grain phenotypes exist. This conclusion is in line with high-heritability estimates and a preponderance of additive inheritance reported early on for TGW in rye (Wolski et al., 1972). Subsequently, major genes responsible for a substantial part of the heritable variance of grain weight in rye have been identified (Wricke, 2002; Skoryk et al., 2010). While the two complementary acting genes *Kernel weight 5* (*KW5*) and *KW7* have been genetically (Wricke, 2002) and physically (Hackauf et al., 2021) mapped on chromosomes 5R and 7R, respectively, map positions of the genes *lg* (large grain) and *tg* (thick grain) identified by Skoryk et al. (2010) are unknown. It is worth noting in this context that there are seven major QTLs identified in the present study on chromosomes 2R, 4R, and 6R, each explaining more than 30% of the phenotypic variance for TGW. Interestingly, one of these QTL explaining 42.7% of the phenotypic variance is located just 3,443 bp upstream of the amino acid transporter SECCE4Rv1G0251940 on chromosome 4R. Most recently, overexpression of the amino acid transporter *TaAAP13* in wheat has significantly increased grain size, grain nitrogen concentration, and thousand-grain weight, indicating that the sink strength for nitrogen transport was increased by manipulation of amino acid transporters (Wan et al., 2021). The large-effect QTL, like those described for TGW and identified for GYD, HDT, GPC, STC, TAX, and WAX, are promising targets for successful marker-assisted selection in rye-improvement programs. Likewise, DNA markers derived from functionally characterized sequence motifs explaining part of the genetic variance have been shown to improve the predictive ability of genomic selection models in plant breeding (Spindel et al., 2016; Bian and Holland, 2017; Liu et al., 2019; Rice and Lipka, 2019). The present study reports SNP markers in candidate regions and genes for nine agronomic and quality traits of rye identified by GWAS methods. The distribution of the cross-validated SNPs indicates no accumulation of SNPs within short Mbp ranges, except for few loci governing TGW on 7R or PHT on 2R. This observation is in line with the expectation for a cross-fertilizing species with a fast decline of LD.

We dissected trait correlations at the gene level and identified a subset of cross-validated SNP on protein-coding sequences associated with more than one trait. Genetic trait correlations might result either from pleiotropy or linkage disequilibrium. The predominant genetic basis of trait correlations is controversial and comprehensively reviewed by Chen and Lübberstedt (2010). The genetic diversity in the protein-coding sequences of the ‘Lo7' genome assembly described in the present study provides candidate genes to further dissect the associated trait correlations based on dedicated genetic and genomic approaches.

### Conserved Genetic Architecture for Complex Traits in Rye and Rice

Rice was the first sequenced crop genome, paving the way for the sequencing of additional and more complex genomes within the grass family (Jackson, 2016). Along with large-scale high-throughput genome-sequencing projects (Wang et al., 2018), rice genomics advanced our understanding of molecular mechanisms-controlling agronomic traits (Li et al., 2018; Song et al., 2018; Yao et al., 2018) and pioneered the direct transfer of basic research to field applications (Wang and Li, 2019; Wang et al., 2021). The common evolutionary origin of the grasses (Pont et al., 2019) served to make use of the rice genome sequence as a blueprint for marker development in rye (Hackauf and Wehling, 2005; Hackauf et al., 2012, 2017b). High-throughput transcript mapping, chromosome survey sequencing, and integration of conserved synteny information of model grass genomes identified 17 conserved syntenic linkage blocks, making up the rye genome in comparison to model grass genomes, including rice (Martis et al., 2013). In wheat and barley, yield-related genes have been identified based on their orthologous genes in rice [*cv*. Nadolska-Orczyk et al. (2017) for review]. Most recently, 237 orthologs of cloned rice QTL have been reported as candidate genes for yield and yield-related traits in a Meta-QTL (MQTL) analysis in bread wheat (Yang et al., 2021). BLASTP sequence similarity searches revealed that neither of the 237 wheat genes correspond to any of the ‘Lo7' gene models associated with yield and yield-related traits in rye. This observation may be attributed to the relatively long linkage disequilibrium decay distance of wheat and a considered co-localization of associated markers obtained from GWAS and an MQTL within a 5-Mb physical region (Yang et al., 2021). The relevance of rice as a model crop for agronomic important traits in grasses is further emphasized by comparative genomic analyses that identified ortho-MQTL at co-linear regions between rice, barley, and maize, respectively (Khahani et al., 2020). The comparative analysis between rye and rice for similar or homologous traits conducted in the present study identified a conserved genetic architecture for agronomic traits that served as cross-species validation of individual MTAs. As a consequence, the commonality between the quantitative trait physiology and the biochemical function of a gene improves our understanding of the molecular nature of QTL in rye and extends our knowledge about causal quantitative trait gene(s) (QTGs) in complex cereal genomes. Given a close evolutionary relationship among grass genomes (Pont et al., 2019), the genomic resources that have been developed for rye (Bauer et al., 2017; Li G. et al., 2021; Rabanus-Wallace et al., 2021) in combination with options of sophisticated experimental designs offered by hybrid rye breeding (Supplementary Figure 1) enable a systematic evaluation of the rich genetic diversity of rye in orthologs of cloned rice QTL for the discovery of gene function to further advance genomics-assisted Triticeae improvement.

### SMART Breeding for Ergot Defense in Rye

The GWAS described in the present study offers novel options for the selection with markers and advanced reproductive technology (SMART) breeding (Davis et al., 1997) to promote the genetic improvement of rye in terms of high yield potential and minimized risk of ergot infestation. Because of the toxicity of ergot sclerotia for humans and animals, the European Commission (EC) Regulation (EU) 2021/1399 amending Regulation (EC) No. 1881/2006 further lowers maximum levels of ergot sclerotia and ergot alkaloids in rye and rye-milling products to 0.2 g/kg as from 1.7.2024. The inclusion of ergot reaction in the German national listing trials is attributed to the genetic diversity of winter rye cultivars in their susceptibility to ergot (Miedaner et al., 2010) and motivated the development of cultivars with improved ergot defense. CMS-based hybrids with an unsatisfactory restoration level and reduced pollen shedding are notably susceptible to ergot as the fungal spores have no competitors during the infection of the stigmatic tissue (Hackauf et al., 2012, 2017b). Restorer-of-fertility (*Rf* ) genes are of central importance for cereal hybrid breeding, both for minimizing ergot infestation (Miedaner et al., 2010), as well as achieving maximum seed setting (Whitford et al., 2013). Indeed, P-type rye hybrids carrying an effective *Rfp* gene suffer from a significant reduction in grain yield (Miedaner et al., 2017). As a consequence of the high yield penalty, a restricted integration of restorer genes like *Rfp1* from weedy rye (Hackauf et al., 2012) in the pollinator gene pool, gaining a restorer index of ~50%, is considered as a feasible practice (Miedaner et al., 2017). However, it needs to be considered that rainy weather at a flowering time reduces pollen shedding and pollen movement. As wet pollen agglutinates and distributes over short distances only, a restorer index of ~50% may result in insufficient quantities of pollen to combat the fungus adequately. In order to comprehensively reduce the risk of ergot infection in hybrid rye, varieties should be developed with a restorer index of 100%, i.e., restoration of male fertility in every single plant of hybrid rye. This strategy is the key to support short-distance pollen distribution, as hybrid rye is able to set seeds upon self-pollination, just like wheat or barley. The associations between genetic causes of phenotypic variation in yield and yield components identified in ‘Lo7' gene models in the present study enhance marker-assisted approaches to improve the ergot defense of rye that is currently solely focused on quick and accurate tracking of *Rfp* genes (Hackauf et al., 2012, 2017b). Knowledge of major QTL-controlling TGW like that residing in close proximity of the amino acid-transporter SECCE4Rv1G0251940 offers a chance to precisely assess natural genetic diversity and counterbalance linkage drag effects of effective *Rfp* genes, as TGW counts among the traits negatively affected by *Rfp* genes (Miedaner et al., 2017). SNPs identified in ‘Lo7' gene models-encoding proteins with a crucial role in plant development like SECCE3Rv1G0201750 or SECCE7Rv1G0479810 provide valuable means for this purpose as well. A particularly attractive target to counterbalance fitness costs of *Rfp* genes measurable as inferior performance in GYD is SECCE3Rv1G0183650. SECCE3Rv1G0183650 is the ortholog of *OsDWT1*, a WUSCHEL-related homeobox (WOX) transcription factor that promotes tiller growth downstream of *SLR1* in rice (Wang et al., 2014). The number of tillers produced per plant is controlled by the environment during the period of tiller development from the three-leaf stage to jointing and the amount of tiller mortality that occurs from jointing to anthesis (Shaaf et al., 2019; Tilley et al., 2019). Recently, empirical data have expanded our understanding of the physiological mechanisms underpinning the yield response to plant density. While a high tillering potential reduces the agronomic optimum plant density in both high and low yield environments, at per-plant scale, a compensation between heads per plant and kernels per head was the main factor contributing to yield with different tillering potentials under varying yield environments (Bastos et al., 2020). Knowledge of genes like SECCE3Rv1G0183650 and the cross-validation strategy described before (Supplementary Figure 1) further advances our knowledge of this critical yield component in order to develop rye varieties with an optimal and environmentally stable tillering potential.

### Advancing Rye to an Authentic High-Performance Crop With Diverse End-Use

Arabinoxylans are non-starch polysaccharides and the predominant components within the endosperm cell walls in rye and, to a lesser degree, in wheat (Buksa et al., 2016; Freeman et al., 2016; Oest et al., 2020). High AX content increases the falling number, dough yield, bread volume, and bread shelf life (Buksa et al., 2010; Oest et al., 2020). Current methods of rye breeding and the growth under severe drought conditions in a changing climate are thought to negatively influence bread qualities, which demands improved understanding of the mechanisms by which proteins, starch, and AX—the most prominent hemicelluloses—might interact (Oest et al., 2020). To increase the value of rye as livestock feed, a low-WAX content is currently considered as a desired grain phenotype (Kobylyansky et al., 2019), in sharp contrast to the optimal needs for bread making (Buksa et al., 2010; Oest et al., 2020). Interestingly, recent research has provided a novel momentum concerning the value of rye AXs for pig feeding. Indeed, beneficial changes in the physicochemical characteristics of digesta of young pigs due to increased rye levels in the diet have been attributed to the very high content of AXs as the predominant “dietary fiber” content of rye, which is beneficial for improving “gut health,” an important parameter in terms of animal health, animal welfare, and food safety (Wilke et al., 2021). In any case, the evaluation of end-use quality parameters like WAX asks for molecular markers that are currently not available for large-scale genotyping of WAX in rye. Our cross-species validation did neither for TAX nor WAX result in the identification of obvious candidate genes. This observation might refer to higher selection pressure on genes controlling these grain-quality parameters in rye or a lack of synteny in rye and rice. However, cross-population validation identified 31 and 15 protein-coding genes, respectively, that deserve a more detailed examination. Remarkably, more than 30% of the cross-validated protein-coding genes associated with WAX are predicted to encode protein kinases, including receptor protein kinases. Receptor protein kinases are discussed to sense cell-wall perturbations originating from osmotic stress (Zhu, 2016). Stress upregulates the expression of expansins and xyloglucan-modifying enzymes that can remodel cell walls (Tenhaken, 2015). Notably, we identified an association of SECCE6Rv1G0407620, encoding a Xyloglucan alpha-1,6-xylosyltransferase and WAX. Most interestingly, we identified major effects (R^2^ > 10%) of a SNP in the receptor protein kinase-like gene model SECCE4Rv1G0223400 on WAX. This observation supports previous research highlighting the impact of receptor protein kinases function in stress responses (Marshall et al., 2012). Further studies will demonstrate if SECCE4Rv1G0223400 is the supposed key regulator of WAX that contributes to improving performance of rye under drought stress. Likewise, in-depth analysis of SECCE4Rv1G0223400 for functional SNPs controlling WAX content depicts an innovative example for SMART breeding of high-quality feed rye varieties with stable contents of WAX. The identification of natural genetic diversity controlling these grain quality traits is particularly important for commercial rye breeding, as the ability to substantially increase TAX and WAX appears a crucial adaptation strategy to drought stress in rye. The knowledge gained in the present study is consistent with previous research in wheat, reporting on increased concentration of AX upon drought stress (Hong et al., 1989; Coles et al., 1997; Gebruers et al., 2010; Rakszegi et al., 2014). AXs are the dominant non-cellulosic polysaccharides in the thick aleurone cell walls in cereal grains, and the second most abundant component in the starchy endosperm cell walls after (1,3;1,4)-β-glucan (Rosicka-Kaczmarek et al., 2016; Hassan et al., 2017). The increase in the dietary fiber AX in rye and wheat under drought stress conditions contributes to remodeling the cell wall composition as a strategy in response to abiotic stress (Tenhaken, 2015). In barley and wheat, AX constitutes 4.2–9.6% and 4.1–9% of grain dry matter, respectively (Martinant et al., 1999; Izydorczyk and Dexter, 2008; Andersson et al., 2009). The effective tolerance of rye toward drought stress (Schittenhelm et al., 2014) is mirrored by the largest amounts of grain AX among cultivated Triticeae species, ranging from 8 to 12.1% (Rosicka-Kaczmarek et al., 2016). As efficient responses to purifying selection as well as significant genetic gains in agronomic traits were feasible not before hybrid breeding started 50 years ago (Laidig et al., 2017), high AX content of rye grains could evolve due to long-lasting natural selection of random mating rye populations in harsh European environments north of the alps on poor, podsolic soils. The significant (*p* < 0.001) negative correlation between GYD and WAX in 2011 refers to a changed carbon allocation and metabolism that resulted in energy dissipation in terms of declined yield. Recent progress in developing random mating feeding rye with low content of WAX (Kobylyansky et al., 2019) has demonstrated that this relationship can be overcome by breeding. For the selection of genotypes with low content of WAX in Central European rye breeding programs, line *per se* performance in well-defined drought stress conditions of rain-out shelters appears a promising strategy due to lower costs of seed production, the higher selection intensity, and the larger proportion of additive genetic variance exploited in inbred lines as compared to hybrids (Miedaner et al., 2014). The significant but weak phenotypic correlation *r*_*P*_ = 0.25–0.28 between line *per se* and testcross performance reported for WAX (Miedaner et al., 2014) probably refers to the different environmental conditions of field experiments conducted by Miedaner and co-workers in 2010 and 2011 and should, thus, not compromise the proposed selection strategy. Levels of precipitation were higher, and average temperature was lower in 2010 as compared to the long-term mean [DWD (Deutscher Wetterdienst), 2010] and the already described natural drought stress in 2011. The cross-validated SNP markers identified in the present study provide essential targets for further research to overcome the strong impact of the environment on TAX and WAX by an efficient and accurate selection of suitable genotypes for the development of rye with certified grain qualities.

### Conclusions

Despite formidable achievements, major challenges in rye production remain. Breeding of cultivars with high yield potential, strong ergot defense, and tailor-made grain qualities is inevitable to further advance rye from an all-rounder to an authentic high-performance crop with different and certified types of end-use. For this purpose, further progress in rye phenomics and functional genomics research is necessary to associate genome sequence information with phenotypes related to rye growth and development. The present study reports candidate regions and genes in the recently published ‘Lo7' high-quality genome assembly (Rabanus-Wallace et al., 2021) for nine agronomic and quality traits of rye identified by GWAS methods as a crucial step to make previously hidden genetic variation accessible to genetic studies and breeding of rye. The observed rich genetic diversity of elite rye germplasm, together with a bulked segregant phenotyping strategy of testcross performance in multi-environmental field trials, supports previous arguments (Hackauf et al., 2017a) for a stronger utilization of rye in research directed to the identification of valuable alleles for Triticeae improvement programs. To conclude, the genomic data generated in this study improve our understanding of the allelic variation in rye germplasm collections and will facilitate the advancing of genomics-assisted rye breeding for variety improvement as well.

## Data Availability Statement

The original contributions presented in the study are included in the article/Supplementary Material, further inquiries can be directed to the corresponding author.

## Author Contributions

The work presented here was carried out in collaboration between all the authors. BH and FF defined the research theme. BH conceived the design of this study, coordinated the experiments, supervised the project, and performed comparative analyses. FF developed plant materials, conducted field trials, and performed the phenotyping of agronomic traits, including grain yield. GJ assessed grain-quality parameters. AK conducted genotyping. BH and DS established the genetic linkage map. DS conducted the GWAS and the analyses of population genetic data. BH, DS, AK, and AZ discussed analyses and interpretation of results. BH wrote and DS, AZ, as well as AK, reviewed and edited the manuscript. All the authors have read and approved the final version of the manuscript.

## Funding

This study was financially supported by the German Federal Ministry of Food and Agriculture based on the decision of the Parliament of the Federal Republic of Germany through the Fachagentur Nachwachsende Rohstoffe e. V. (Grant Nos. 22014509 and 22005910). We gratefully acknowledge support by the Open Access Publication Funds of the Julius Kühn-Institute.

## Conflict of Interest

DS and FF are employed by HYBRO Saatzucht GmbH & Co. KG, and AK is employed by Diversity Arrays Technologies. The companies have a commercial interest in the results for application in variety development and for the provision of molecular genetic services. This does not alter the adherence of the authors to all Frontiers in Plant Science policies on sharing data and materials. The remaining authors declare that the research was conducted in the absence of any commercial or financial relationships that could be construed as a potential conflict of interest.

## Publisher's Note

All claims expressed in this article are solely those of the authors and do not necessarily represent those of their affiliated organizations, or those of the publisher, the editors and the reviewers. Any product that may be evaluated in this article, or claim that may be made by its manufacturer, is not guaranteed or endorsed by the publisher.

## Supplementary Material

Supplementary Figure 1 Flow diagram illustrating seed production of near-isogenic experimental rye hybrids for cross-validation of candidate genes by bulked segregant phenotyping. P1, P2: parental genotypes, M, m: superior and inferior allele at a given SNP marker.

## References

[B1] Agelet L. E. Hurburgh C. R. Jr. (2010). A tutorial on near infrared spectroscopy and its calibration. Crit. Rev. Anal. Chem. 40, 246–260. 10.1080/10408347.2010.515468

[B2] Alqudah A. M. Sallam A. Baenziger S. P. Börner A. (2019). GWAS: fast-forwarding gene identification and characterization in temperate Cereals: lessons from Barley - a review. J. Adv. Res. 22, 119–135. 10.1016/j.jare.2019.10.013 31956447 PMC6961222

[B3] Altschul S. F. Gish W. Miller W. Myers E. W. Lipman D. J. (1990). Basic local alignment search tool. J. Mol. Biol. 215, 403–410. 10.1016/S0022-2836(05)80360-2 2231712

[B4] Andersson R. Fransson G. Tietjen M. Åman P. (2009). Content andmolecular-weight distribution of dietary fiber components inwhole-grain rye flour and bread. J. Agric. Food Chem. 57, 2004–2008. 10.1021/jf801280f 19219994

[B5] Auinger H. J. Schönleben M. Lehermeier C. Schmidt M. Korzun V. Geiger H. H.. (2016). Model training across multiple breeding cycles significantly improves genomic prediction accuracy in rye (*Secale cereale* L.). Theor. Appl. Genet. 129, 2043–2053. 10.1007/s00122-016-2756-5 27480157 PMC5069347

[B6] Bastos L. M. Carciochi W. Lollato R. P. Jaenisch B. R. Rezende C. R. Schwalbert R.. (2020). Winter wheat yield response to plant density as a function of yield environment and tillering potential: a review and field studies. Front. Plant Sci. 11:54. 10.3389/fpls.2020.00054 32194579 PMC7066254

[B7] Bauer E. Schmutzer T. Barilar I. Mascher M. Gundlach H. Martis M. M.. (2017). Towards a whole-genome sequence for rye (*Secale cereale* L.) Plant J. 89, 853–869. 10.1111/tpj.13436 27888547

[B8] Beillouin D. Schauberger B. Bastos A. Ciais P. Makowski D. (2020). Impact of extreme weather conditions on European crop production in 2018. Philos. Trans. R. Soc. Lond. B. Biol. Sci. 375:20190510. 10.1098/rstb.2019.0510 32892735 PMC7485097

[B9] Bellucci A. Tondelli A. Fangel J. U. Torp A. M. Xu X. Willats W. G.. (2017). Genome-wide association mapping in winter barley for grain yield and culm cell wall polymer content using the high-throughput CoMPP technique. PLoS ONE 12:e0173313. 10.1371/journal.pone.0173313 28301509 PMC5354286

[B10] Benjamini Y. Hochberg Y. (1995). Controlling the false discovery rate: a practical and powerful approach to multiple testing. J. Royal Statistical Soc. 57, 289–300. 10.1111/j.2517-6161.1995.tb02031.x

[B11] Bevan M. W. Uauy C. Wulff B. B. Zhou J. Krasileva K. Clark M. D. (2017). Genomic innovation for crop improvement. Nature 543, 346–354. 10.1038/nature22011 28300107

[B12] Bian Y. Holland J. B. (2017). Enhancing genomic prediction with genome-wide association studies in multiparental maize populations. Heredity 118:585. 10.1038/hdy.2017.4 28198815 PMC5436027

[B13] Bilgrami S. S. Ramandi H. D. Shariati V. Razavi K. Tavakol E. Fakheri B. A.. (2020). Detection of genomic regions associated with tiller number in Iranian bread wheat under different water regimes using genome-wide association study. Sci. Rep. 10:14034. 10.1038/s41598-020-69442-9 32820220 PMC7441066

[B14] Blum A. Golan G. Mayer J. Sinmena B. (1997). The effect of dwarfing genes on sorghum grain filling from remobilized stem reserves, under stress. Field Crops Res. 52, 43–54. 10.1016/S0378-4290(96)03462-4

[B15] Boeven P. Zhao Y. Thorwarth P. Liu F. Maurer H. P. Gils M.. (2020). Negative dominance and dominance-by-dominance epistatic effects reduce grain-yield heterosis in wide crosses in wheat. Sci. Adv. 6:eaay4897. 10.1126/sciadv.aay4897 32582844 PMC7292627

[B16] Bolibok-Bragoszewska H. Heller-Uszyńska K. Wenzl P. Uszyński G. Kilian A. Rakoczy-Trojanowska M. (2009). DArT markers for the rye genome - genetic diversity and mapping. BMC Genomics 10:578. 10.1186/1471-2164-10-578 19958552 PMC2795769

[B17] Börner A. Melz G. (1988). Response of rye genotypes differing in plant height to exogenous gibberellic acid application. Arch. Züchtungsforsch 18, 71–74.

[B18] Borzecka E. Hawliczek-Strulak A. Bolibok L. Gawroński P. Tofil K. Milczarski P. (2018). Effective BAC clone anchoring with genotyping-by-sequencing and Diversity Arrays Technology in a large genome cereal rye. Sci. Rep. 8:8428. 10.1038/s41598-018-26541-y 29849048 PMC5976670

[B19] Bradbury P. J. Zhang Z. Kroon D. E. Casstevens T. M. Ramdoss Y. Buckler E. S. (2007). TASSEL: software for association mapping of complex traits in diverse samples. Bioinformatics 23, 2633–2635. 10.1093/bioinformatics/btm308 17586829

[B20] Braun E. M. Tsvetkova N. Rotter B. Siekmann D. Schwefel K. Krezdorn N.. (2019). Gene expression profiling and fine mapping identifies a gibberellin 2-oxidase gene co-segregating with the dominant dwarfing gene *Ddw1* in Rye (*Secale cereale* L.). Front. Plant Sci. 10:857. 10.3389/fpls.2019.00857 31333700 PMC6616298

[B21] Brinton J. Ramirez-Gonzalez R. H. Simmonds J. Wingen L. Orford S. Griffiths S. (2020). A haplotype-led approach to increase the precision of wheat breeding. Commun Biol. 3:712. 10.1038/s42003-020-01413-2 33239669 PMC7689427

[B22] Browning B. L. Yu Z. (2009). Simultaneous genotype calling and haplotype phasing improves genotype accuracy and reduces false-positive associations for genome-wide association studies. Am. J. Hum. Genet. 85, 847–861. 10.1016/j.ajhg.2009.11.004 19931040 PMC2790566

[B23] Brzyski D. Peterson C. B. Sobczyk P. Candès E. J. Bogdan M. Sabatti C. (2017). Controlling the rate of GWAS false discoveries. Genetics 205, 61–75. 10.1534/genetics.116.193987 27784720 PMC5223524

[B24] Buksa K. Nowotna A. Praznik W. Gambuś H. Ziobro R. Krawontka J. (2010). The role of pentosans and starch in baking of wholemeal rye bread. Food Res. Intern. 43, 2045–2051. 10.1016/j.foodres.2010.06.005

[B25] Buksa K. Praznik W. Loeppert R. Nowotna A. (2016). Characterization of water and alkali extractable arabinoxylan from wheat and rye under standardized conditions. J. Food Sci. Technol. 53, 1389–1398. 10.1007/s13197-015-2135-2 27570263 PMC4984697

[B26] Bundessortenamt (2013). Beschreibende Sortenliste Getreide, Mais, Öl- und Faserpflanzen, Leguminosen, Rüben und Zwischenfrüchte 2013. Hannover.

[B27] Cavalli-Sforza L. L. Edwards A. W. (1967). Phylogenetic analysis. Models and estimation procedures. Am. J. Hum. Genet. 19, 233–257. 6026583 PMC1706274

[B28] Chen Y. Lübberstedt T. (2010). Molecular basis of trait correlations. Trends Plant Sci. 15, 454–461. 10.1016/j.tplants.2010.05.004 20542719

[B29] Coles G. D. Hartunian-Sowa S. M. Jamieson P. D. Hay A. J. Atwell W. A. Fulcher R. G. (1997). Environmentally-induced variation in starch and non-starch polysaccharide content in wheat. J. Cereal Sci. 26, 47–54. 10.1006/jcrs.1996.0102

[B30] Comadran J. Thomas W. T. van Eeuwijk F. A. Ceccarelli S. Grando S. Stanca A. M.. (2009). Patterns of genetic diversity and linkage disequilibrium in a highly structured *Hordeum vulgare* association-mapping population for the Mediterranean basin. Theor. Appl. Genet. 119, 175–187. 10.1007/s00122-009-1027-0 19415228

[B31] Davis G. P. D'occhio M. J. Hetzel D. J. S. (1997). “SMART breeding: selection with markers and advanced reproductive technologies,” in Proceedings of the 12th Conference of the Association for the Advancement of Animal Breeding and Genetics (Dubbo, NSW), 429–432.

[B32] Dill P. (1983). Zur züchterischen Verbesserung der Kornmasse bei Winterroggen (*Secale cereale* L.). Arch. Züchtungsforsch 13, 157–168.

[B33] Dill P. (1989). Zur züchterischen Verbesserung der Kornmasse bei Winterroggen (*Secale cereale* L.) – Ergebnisse von Drillprüfungen. Arch. Züchtungsforsch 20, 329–337.

[B34] Dittmer H. J. (1937). A quantitative study of the roots and root hairs of a winter rye plant (*Secale cereale*). Am. J. Bot. 24, 417–420. 10.1002/j.1537-2197.1937.tb09121.x

[B35] DWD (Deutscher Wetterdienst). (2010). Jahresrückblick: Deutschlandwetter im Jahr 2010. Available online at: https://www.dwd.de/DE/presse/pressemitteilungen/DE/2010/20101229_deutschlandwetter_jahr.pdf?__blob=publicationFile&v=3

[B36] DWD (Deutscher Wetterdienst) (2012). Drought Conditions in Europe in the Spring of 2012. Available online at: https://www.dwd.de/EN/ourservices/specialevents/drought/20120810_Trockenheit_2012_en.pdf?__blob=publicationFile&v=4

[B37] Evanno G. Regnaut S. Goudet J. (2005). Detecting the number of clusters of individuals using the software STRUCTURE: a simulation study. Mol. Ecol. 14, 2611–2620. 10.1111/j.1365-294X.2005.02553.x 15969739

[B38] Excoffier L. Smouse P. E. Quattro J. M. (1992). Analysis of molecularvariance inferred from metric distances among DNA haplotypes:application to human mitochondrial DNA restrictiondata. Genetics 131, 479–491. 10.1093/genetics/131.2.479 1644282 PMC1205020

[B39] FAOSTAT (2020). Production. Crops. “Rye”, “World.” Rome: FAO. Available online at: http://www.fao.org/faostat/en/#data/QC (accessed August 25, 2021).

[B40] Finno C. J. Aleman M. Higgins R. J. Madigan J. E. Bannasch D. L. (2014). Risk of false positive genetic associations in complex traits withunderlying population structure: a case study. Vet. J. 202, 543–549. 10.1016/j.tvjl.2014.09.013 25278384 PMC4337777

[B41] Freeman J. Lovegrove A. Wilkinson M. D. Saulnier L. Shewry P. R. Mitchell R. A. (2016). Effect of suppression of arabinoxylan synthetic genes in wheat endosperm on chain length of arabinoxylan and extract viscosity. Plant Biotechnol. J. 14, 109–116. 10.1111/pbi.12361 25819752 PMC5098169

[B42] Gaikpa D. S. Koch S. Fromme F. J. Siekmann D. Würschum T. Miedaner T. (2020). Genome-wide associa-tion mapping and genomic prediction of Fusarium head blight resistance, heading stage and plant height in winter rye (*Secale cereale*). Plant Breed. 139, 508–520. 10.1111/pbr.12810

[B43] Gao L. Meng C. Yi T. Xu K. Cao H. Zhang S.. (2021). Genome-wide association study reveals the genetic basis of yield- and quality-related traits in wheat. BMC Plant Biol. 21:144. 10.1186/s12870-021-02925-7 33740889 PMC7980635

[B44] Gebruers K. Dornez E. Bedõ Z. Rakszegi M. Frás A. Boros D.. (2010). Environment and genotype effects on the content of dietary fiber and its components in wheat in the HEALTHGRAIN diversity screen. J. Agric. Food Chem. 58, 9353–9361. 10.1021/jf100447g 20462191

[B45] Geiger H. H. Miedaner T. (2009). “Rye breeding,” in Cereals, Handbook of Plant Breeding, Vol. 3, ed M. J. Carena (New York, NY: Springer Science + Business Media), 157–181.

[B46] Geiger H. H. Schnell F. W. (1970). Cytoplasmic male sterility in rye (*Secale cereale* L.). Crop Sci. 10:590–593. 10.2135/cropsci1970.0011183X001000050043x24196055

[B47] Gruner P. Schmitt A. K. Flath K. Piepho H. P. Miedaner T. (2021). Mapping and validating stem rust resistance genes directly in self-incompatible genetic resources of winter rye. Theor. Appl. Genet. 134, 1989–2003. 10.1007/s00122-021-03800-7 33688982 PMC8263455

[B48] Hackauf B. Bauer E. Korzun V. Miedaner T. (2017b). Fine mapping of the restorer gene *Rfp3* from an Iranian primitive rye (*Secale cereale* L.). Theor. Appl. Genet. 130, 1179–1189. 10.1007/s00122-017-2879-3 28315925

[B49] Hackauf B. Haffke S. Fromme F. J. Roux S. R. Kusterer B. Musmann D.. (2017a). QTL mapping and comparative genome analysis of agronomic traits including grain yield in winter rye. Theor. Appl. Genet. 130, 1801–1817. 10.1007/s00122-017-2926-0 28567664

[B50] Hackauf B. Korzun V. Wortmann H. Wilde P. Wehling P. (2012). Development of conserved ortholog set markers linked to the restorer gene *Rfp1* in rye. Mol. Breed. 30, 1507–1518. 10.1007/s11032-012-9736-5

[B51] Hackauf B. Rabanus-Wallace M. T. Korzun V. (2021). “Bridging the genotype-phenotype gap for precision breeding in rye,” in The Rye Genome, 1st Edn, eds N. Stein, M. T. Rabanus-Wallace, and C. Kole (Compendium of Plant Genomes; Springer International Publishing), 46. Available online at: https://www.springer.com/de/book/9783030833824

[B52] Hackauf B. Rudd S. van der Voort J. R. Miedaner T. Wehling P. (2009). Comparative mapping of DNA sequences in rye (*Secale cereale* L.) in relation to the rice genome. Theor. Appl. Genet. 118, 371–384. 10.1007/s00122-008-0906-0 18953524

[B53] Hackauf B. Wehling P. (2005). Approaching the self-incompatibility locus *Z* in rye (*Secale cereale* L.) via comparative genetics. Theor. Appl. Genet. 110, 832–845. 10.1007/s00122-004-1869-4 15717193

[B54] Hagenblad J. Oliveira H. R. Forsberg N. E. Leino M. W. (2016). Geographical distribution of genetic diversity in Secale landrace and wild accessions. BMC Plant Biol. 16:23. 10.1186/s12870-016-0710-y 26786820 PMC4719562

[B55] Han Y. Broughton S. Liu L. Zhang X. Q. Zeng J. He X.. (2020). Highly efficient and genotype-independent barley gene editing based on anther culture. Plant Commun. 2:100082. 10.1016/j.xplc.2020.100082 33898972 PMC8060703

[B56] Hardy O. J. (2003). Estimation of pairwise relatedness between individuals and characterization of isolation-by-distance processes using dominant genetic markers. Mol. Ecol. 12, 1577–1588. 10.1046/j.1365-294X.2003.01835.x 12755885

[B57] Hardy O. J. Vekemans X. (2002). SPAGeDi: a versatile computer program to analyse spatial genetic structure at the individual or population levels. Mol. Ecol. Notes 2, 618–620. 10.1046/j.1471-8286.2002.00305.x

[B58] Hassan A. S. Houston K. Lahnstein J. Shirley N. Schwerdt J. G. Gidley M. J.. (2017). A Genome Wide Association Study of arabinoxylan content in 2-row spring barley grain. PLoS ONE 12:e0182537. 10.1371/journal.pone.0182537 28771585 PMC5542645

[B59] Hawliczek A. Bolibok L. Tofil K. Borzecka E. Jankowicz-Cieślak J. Gawroński P.. (2020). Deep sampling and pooled amplicon sequencing reveals hidden genic variation in heterogeneous rye accessions. BMC Genomics 21:845. 10.1186/s12864-020-07240-3 33256606 PMC7706248

[B60] Hepting L. (1978). Analyse eines 7x7–Sortendiallels zur Ermittlung geeigneten Ausgangsmaterials für die Hybridzüchtung bei Roggen. Z. Pflanzenzüchtung 80, 188–197.

[B61] Hill C. B. Angessa T. T. Zhang X. Q. Chen K. Zhou G. Tan C.. (2021). A global barley panel revealing genomic signatures of breeding in modern Australian cultivars. Plant J. 106, 419–434. 10.1111/tpj.15173 33506596

[B62] Hong B. H. Rubenthaler G. L. Allen R. E. (1989). Wheat pentosans. I. Cultivar variation and relationship to kernel hardness. Cereal Chem. 66, 369–373.

[B63] Huang X. Han B. (2014). Natural variations and genome-wide association studies in crop plants. Ann. Rev. Plant Biol. 65, 531–551. 10.1146/annurev-arplant-050213-035715 24274033

[B64] Hübner M. Wilde P. Schmiedchen B. Dopierala P. Gowda M. Reif J. C.. (2013). Hybrid rye performance under natural drought stress in Europe. Theor. Appl. Genet. 126, 475–482. 10.1007/s00122-012-1994-4 23090142

[B65] Izydorczyk M. S. Dexter J. E. (2008). Barley β-glucans and arabinoxylans: molecular structure, physicochemicalproperties, and uses in food products - a review. Food Res. Intern. 41, 850–868. 10.1016/j.foodres.2008.04.001

[B66] Jackson S. A. (2016). Rice: the first crop genome. Rice 9:14. 10.1186/s12284-016-0087-4 27003180 PMC4803718

[B67] Jamann T. M. Balint-Kurti P. J. Holland J. B. (2015). QTL mapping using high-throughput sequencing. Methods Mol. Biol. 1284, 257–285. 10.1007/978-1-4939-2444-8_13 25757777

[B68] Jayakodi M. Padmarasu S. Haberer G. Bonthala V. S. Gundlach H. Monat C.. (2020). The barley pan-genome reveals the hidden legacy of mutation breeding. Nature 588, 284–289. 10.1038/s41586-020-2947-8 33239781 PMC7759462

[B69] Jürgens H. U. Jansen G. Wegener C. B. (2012). Characterisation of several rye cultivars with respect to arabinoxylans and extract viscosity. J. Agricult. Sci. 4, 1–12. 10.5539/jas.v4n5p1

[B70] Kempthorne O. (1956). The theory of the diallel cross. Genetics 41, 451–459. 10.1093/genetics/41.4.451 17247640 PMC1209794

[B71] Khahani B. Tavakol E. Shariati V. Fornara F. (2020). Genome wide screening and comparative genome analysis for Meta-QTLs, ortho-MQTLs and candidate genes controlling yield and yield-related traits in rice. BMC Genomics 21:294. 10.1186/s12864-020-6702-1 32272882 PMC7146888

[B72] Kobylyansky V. D. Kuznetsova L. I. Solodukhina O. V. Lavrentyeva N. S. Timina M. A. (2019). Prospects of using low-pentosan grain fodder rye for baking purposes. Russ. Agricult. Sci. 45, 1–4. 10.3103/S1068367419010063

[B73] Kottmann L. Wilde P. Schittenhelm S. (2016). How do timing, duration, and intensity of drought stress affect the agronomic performance of winter rye? Europ. J. Agron. 75, 25–32. 10.1016/j.eja.2015.12.010

[B74] Labroo M. R. Studer A. J. Rutkoski J. E. (2021). Heterosis and hybrid crop breeding: a multidisciplinary review. Front. Genet. 12:643761. 10.3389/fgene.2021.643761 33719351 PMC7943638

[B75] Ladejobi O. Mackay I. J. Poland J. Praud S. Hibberd J. M. Bentley A. R. (2019). Reference genome anchoring of high-density markers for association mapping and genomic prediction in european winter wheat. Front. Plant Sci. 10:1278. 10.3389/fpls.2019.01278 31781130 PMC6857554

[B76] Laidig F. Piepho H. P. Rentel D. Drobek T. Meyer U. Huesken A. (2017). Breeding progress, variation, and correlation of grain and quality traits in winter rye hybrid and population varieties and national on-farm progress in Germany over 26 years. Theor. Appl. Genet. 30, 981–998. 10.1007/s00122-017-2865-9 28289803 PMC5395587

[B77] Lal R. (2016). Global food security and nexus thinking. J. Soil Water Conserv. 71, 85A–90A. 10.2489/jswc.71.4.85A

[B78] Langstroff A. Heuermann M. C. Stahl A. Junker A. (2021). Opportunities and limits of controlled-environment plant phenotyping for climate response traits. Theor. Appl. Genet. 10.1007/s00122-021-03892-1. [Epub ahead of print]. 34302493 PMC8741719

[B79] Łapiński M. Stojałowski S. (2003). Occurrence and genetic identity of male sterility-inducing cytoplasm in rye (*Secale* spp.). Plant Breed. Seed Sci. 48, 7–23.

[B80] Li G. Wang L. Yang J. He H. Jin H. Li X.. (2021). A high-quality genome assembly highlights rye genomic characteristics and agronomically important genes. Nat. Genet. 53, 574–584. 10.1038/s41588-021-00808-z 33737755 PMC8035075

[B81] Li M. Geng L. Xie S. Wu D. Ye L. Zhang G. (2021). Genome-wide association study on total starch, amylose and amylopectin in barley grain reveals novel putative alleles. Int. J. Mol. Sci. 22:553. 10.3390/ijms22020553 33430526 PMC7828029

[B82] Li Y. Haseneyer G. Schön C. C. Ankerst D. Korzun V. Wilde P.. (2011). High levels of nucleotide diversity and fast decline of linkage disequilibrium in rye (*Secale cereale* L.) genes involved in frost response. BMC Plant Biol. 11:6. 10.1186/1471-2229-11-6 21219606 PMC3032657

[B83] Li Y. Xiao J. Chen L. Huang X. Cheng Z. Han B.. (2018). Rice functional genomics research: past decade and future. Mol. Plant. 11, 359–380. 10.1016/j.molp.2018.01.007 29409893

[B84] Liu C. Sukumaran S. Jarquin D. Crossa J. Dreisigacker S. Sansaloni C.. (2020). Comparison of array- and sequencing-based markers for genome-wide association mapping and genomic prediction in spring wheat. Crop Sci. 60, 211–225. 10.1002/csc2.20098

[B85] Liu H. J. Yan J. (2019). Crop genome-wide association study: a harvest of biological relevance. Plant J. 97, 8–18. 10.1111/tpj.14139 30368955

[B86] Liu X. Wang H. Hu X. Li K. Liu Z. Wu Y.. (2019). Improving genomic selection with quantitative trait loci and nonadditive effects revealed by empirical evidence in maize. Front. Plant Sci. 10:1129. 10.3389/fpls.2019.01129 31620155 PMC6759780

[B87] Liu Y. Lin Y. Gao S. Li Z. Ma J. Deng M.. (2017). A genome-wide association study of 23 agronomic traits in Chinese wheat landraces. Plant J. 91:861–873. 10.1111/tpj.13614 28628238

[B88] Loos R. J. F. (2020). 15 years of genome-wide association studies and no signs of slowing down. Nat. Commun. 11:5900. 10.1038/s41467-020-19653-5 33214558 PMC7677394

[B89] Mackay T. F. (2004). The genetic architecture of quantitative traits: lessons from Drosophila. Curr Opin Genet Dev. 14, 253–257. 10.1016/j.gde.2004.04.003 15172667

[B90] Mackay T. F. C. Stone E. A. Ayroles J. F. (2009). The genetics of quantitative traits: challenges and prospects. Nat. Rev. Genet. 10, 565–577. 10.1038/nrg2612 19584810

[B91] Malysheva-Otto L. V. Ganal M. W. Röder M. S. (2004). Analysis of molecular diversity, population structure and linkage disequilibrium in a worldwide survey of cultivated barley germplasm (*Hordeum vulgare* L.). BMC Genet. 7:6. 10.1186/1471-2156-7-6 16433922 PMC1408084

[B92] Marshall A. Aalen R. B. Audenaert D. Beeckman T. Broadley M. R. Butenko M. A.. (2012). Tackling drought stress: receptor-like kinases present new approaches. Plant Cell 24, 2262–2278. 10.1105/tpc.112.096677 22693282 PMC3406892

[B93] Martinant J. P. Billot A. Bouguennec A. Charmet G. Saulnier L. Branlard G. (1999). Genetic and environmental variations inwater-extractable arabinoxylans content and flour extract viscosity. J. Cereal Sci. 30, 45–48. 10.1006/jcrs.1998.0259

[B94] Martis M. M. Zhou R. Haseneyer G. Schmutzer T. Vrána J. Kubaláková M.. (2013). Reticulate evolution of the rye genome. Plant Cell. 25, 3685–3698. 10.1105/tpc.113.114553 24104565 PMC3877785

[B95] Melchinger A. E. (1999). “Genetic diversity and heterosis,” in The Genetics and Exploitation of Heterosis in Crops, eds C. G. Coors and S. Pandey (Madison, WI: American Society of Agronomy), 99–118.

[B96] Melz G. Melz G. Hartman F. (2003). Genetics of a male-sterile rye of 'G-type' with results of the first F1-hybrids. Plant Breed. Seed Sci. 47, 47–55.

[B97] Meng L. Li H. Zhang L. Wang J. (2015). QTL IciMapping: Integrated software for genetic linkage map construction and quantitative trait locus mapping in biparental populations. Crop J. 3, 269–283. 10.1016/j.cj.2015.01.001

[B98] Miedaner T. Haffke S. Siekmann D. Fromme F. J. Roux S. R. Hackauf B. (2018). Dynamic quantitative trait loci (QTL) for plant height predict biomass yield in hybrid rye (*Secale cereale* L.). Biomass Bioenergy 115, 10–18. 10.1016/j.biombioe.2018.04.001

[B99] Miedaner T. Herter C. P. Goßlau H. Wilde P. Hackauf B. (2017). Correlated effects of exotic pollen-fertility restorer genes on agronomic and quality traits of hybrid rye. Plant Breed. 136, 224–229. 10.1111/pbr.12456

[B100] Miedaner T. Hübner M. Korzun V. Schmiedchen B. Bauer E. Haseneyer G.. (2012). Genetic architecture of complex agronomic traits examined in two testcross populations of rye (*Secale cereale* L.). BMC Genomics 13:706. 10.1186/1471-2164-13-706 23244545 PMC3566906

[B101] Miedaner T. Mirdita V. Rodemann B. Drobeck T. Rentel D. (2010). Genetic variation of winter rye cultivars for their ergot (*Claviceps purpurea*) reaction tested in a field design with minimized interplot interference. Plant Breed. 129, 58–62. 10.1111/j.1439-0523.2009.01646.x

[B102] Miedaner T. Schwegler D. D. Wilde P. Reif J. (2014). Association between line *per se* and testcross performance for eight agronomic and quality traits in winter rye. Theor. Appl. Gen. 127, 33–41. 10.1007/s00122-013-2198-2 24072205

[B103] Milczarski P. Hanek M. Tyrka M. Stojałowski S. (2016). The application of GBS markers for extending the dense genetic map of rye (*Secale cereale* L.) and the localization of the *Rfc1* gene restoring male fertility in plants with the C source of sterility-inducing cytoplasm. J. Appl. Genet. 57, 439–451. 10.1007/s13353-016-0347-4 27085345 PMC5061839

[B104] Muqaddasi Q. H. Brassac J. Ebmeyer E. Kollers S. Korzun V. Argillier O.. (2020). Prospects of GWAS and predictive breeding for European winter wheat's grain protein content, grain starch content, and grain hardness. Sci. Rep. 10:12541. 10.1038/s41598-020-69381-5 32719416 PMC7385145

[B105] Nadolska-Orczyk A. Rajchel I. K. Orczyk W. Gasparis S. (2017). Major genes determining yield-related traits in wheat and barley. Theor. Appl. Genet. 130, 1081–1098. 10.1007/s00122-017-2880-x 28314933 PMC5440550

[B106] Nei M. (1987). Molecular Evolutionary Genetics. New York, NY: ColumbiaUniversity Press.

[B107] Oest M. Bindrich U. Voß A. Kaiser H. Rohn S. (2020). Rye bread defects: analysis of composition and further influence factors as determinants of dry-baking. Foods 9:1900. 10.3390/foods9121900 33352657 PMC7765839

[B108] Orr H. A. (1998). The population genetics of adaptation: the distribution of factors fixed during adaptive evolution. Evolution 52, 935–949. 10.1111/j.1558-5646.1998.tb01823.x 28565213

[B109] Paponov I. A. Lebedinskai S. Koshkin E. I. (1999). Growth analysis of solution culture-grown winter rye, wheat and Triticale at different relative rates of nitrogen supply. Ann. Bot. 84, 467–473. 10.1006/anbo.1999.0935

[B110] Pasam R. K. Sharma R. Malosetti M. van Eeuwijk F. A. Haseneyer G. Kilian B.. (2012). Genome-wide association studies for agronomical traits in a world wide spring barley collection. BMC Plant Biol. 12:16. 10.1186/1471-2229-12-16 22284310 PMC3349577

[B111] Pont C. Wagner S. Kremer A. Orlando L. Plomion C. Salse J. (2019). Paleogenomics: reconstruction of plant evolutionary trajectories from modern and ancient DNA. Genome Biol. 20:29. 10.1186/s13059-019-1627-1 30744646 PMC6369560

[B112] Pritchard J. K. Stephens M. Donnelly P. (2000). Inference of population structure using multilocus genotype data. Genetics 155, 945–959. 10.1093/genetics/155.2.945 10835412 PMC1461096

[B113] R Core Team (2017). R: A Language and Environment for Statistical Computing, v.3.4.1 Edn. Vienna: R Foundation for Statistical Computing. Available online at: https://www.r-project.org/

[B114] Rabanus-Wallace M. T. Hackauf B. Mascher M. Lux T. Wicker T. Gundlach H.. (2021). Chromosome-scale genome assembly provides insights into rye biology, evolution, and agronomic potential. Nat. Genet. 53, 564–573. 10.1038/s41588-021-00807-0 33737754 PMC8035072

[B115] Rakoczy-Trojanowska M. Krajewski P. Bocianowski J. Schollenberger M. Wakuliński W. Milczarski P.. (2017). Identification of single nucleotide polymorphisms associated with brown rust resistance, α-amylase activity and pre-harvest sprouting in rye (*Secale cereale* L.). Plant Mol. Biol. Rep. 35, 366–378. 10.1007/s11105-017-1030-6 28603340 PMC5443880

[B116] Rakszegi M. Lovegrove A. Balla K. Láng L. Bedo Z. Veisz O.. (2014). Effect of heat and drought stress on the structure and composition of arabinoxylan and β-glucan in wheat grain. Carbohydr. Polym. 102, 557–565. 10.1016/j.carbpol.2013.12.005 24507319

[B117] Remington D. L. Purugganan M. D. (2003). Candidate genes, quantitative trait loci, and functional trait evolution in plants. Int. J. Plant Sci. 164(3 Suppl.), S7–S20. 10.1086/367812

[B118] Remington D. L. Thornsberry J. M. Matsuoka Y. Wilson L. M. Whitt S. R. Doebley J.. (2001). Structure of linkage disequilibrium and phenotypic associations in the maize genome. Proc. Natl. Acad. Sci. U.S.A. 98, 11479–11484. 10.1073/pnas.201394398 11562485 PMC58755

[B119] Reynolds M. Chapman S. Crespo-Herrera L. Molero G. Mondal S. Pequeno D. N. L.. (2020). Breeder friendly phenotyping. Plant Sci. 295:110396. 10.1016/j.plantsci.2019.110396 32534615

[B120] Rice B. Lipka A. E. (2019). Evaluation of RR-BLUP genomic selection models that incorporate peak genome-wide association study signals in maize and sorghum. Plant Genome 12, 1–14. 10.3835/plantgenome2018.07.0052 30951091 PMC12962346

[B121] Rieseberg L. H. Blackman B. K. (2010). Speciation genes in plants. Ann. Bot. 106, 439–455. 10.1093/aob/mcq126 20576737 PMC2924826

[B122] Rode J. Ahlemeyer J. Friedt W. Ordon F. (2012). Identification of marker-trait associations in the German winter barley breeding gene pool (*Hordeum vulgare* L.). Mol. Breed. 30, 831–843. 10.1007/s11032-011-9667-6

[B123] Roff D. A. (2007). A centennial celebration for quantitative genetics. Evolution 61, 1017–1032. 10.1111/j.1558-5646.2007.00100.x 17492957

[B124] Rosicka-Kaczmarek J. Komisarczyk A. Nebesny E. Makowski B. (2016). The influence of arabinoxylans on the quality of grain industryproducts. Eur. Food Res. Technol. 242, 295–303. 10.1007/s00217-015-2549-0

[B125] Sansaloni C. Franco J. Santos B. Percival-Alwyn L. Singh S. Petroli C.. (2020). Diversity analysis of 80,000 wheat accessions reveals consequences and opportunities of selection footprints. Nat. Commun. 11:4572. 10.1038/s41467-020-18404-w 32917907 PMC7486412

[B126] Schils R. Olesen J. E. Kersebaum K.-C. Rijka B. Oberforster M. Kalyada V.. (2018). Cereal yield gaps across Europe. Europ. J. Agron. 101, 109–120. 10.1016/j.eja.2018.09.003

[B127] Schittenhelm S. Kraft M. Wittich K. P. (2014). Performance of winter cereals grown on field-stored soil moisture only. Eur. J. Agron. 52, 247–258. 10.1016/j.eja.2013.08.010

[B128] Schreiber M. Himmelbach A. Börner A. Mascher M. (2018). Genetic diversity and relationship between domesticated rye and its wild relatives as revealed through genotyping-by-sequencing. Evol. Appl. 12, 66–77. 10.1111/eva.12624 30622636 PMC6304746

[B129] Semagn K. Babu R. Hearne S. (2014). Single nucleotide polymorphism genotypingusing Kompetitive Allele Specific PCR (KASP): Over view of the technology andits application in crop improvement. Mol. Breed. 33, 1–14. 10.1007/s11032-013-9917-x

[B130] Shaaf S. Bretani G. Biswas A. Fontana I. M. Rossini L. (2019). Genetics of barley tiller and leaf development. J. Integr. Plant Biol. 61, 226–256. 10.1111/jipb.12757 30548413

[B131] Sidhu J. S. Ramakrishnan S. M. Ali S. Bernardo A. Bai G. Abdullah S.. (2019). As-sessing the genetic diversity and characterizing genomic regions conferring Tan Spot resistance in cultivated rye. PLoS ONE. 14:e0214519. 10.1371/journal.pone.0214519 30921415 PMC6438500

[B132] Skoryk V. V. Skoryk V. V. Simonenko N. V. Skoryk O. P. (2010). Genetics characteristics of the donor for dominant short stem and large grain winter rye (*Secale cereale* L.). Plant Var. Stud. Prot. J. Appl. Res. 1, 5–12. 10.21498/2518-1017.1(11)0.2010.59362

[B133] Song S. Tian D. Zhang Z. Hu S. Yu J. (2018). Rice genomics: over the past two decades and into the future. Genomics Proteomics Bioinformatics 16, 397–404. 10.1016/j.gpb.2019.01.001 30771506 PMC6411948

[B134] Spindel J. Begum H. Akdemir D. Collard B. Redoña E. Jannink J.-L. (2016). Genome-wide prediction models that incorporate *de novo* GWAS are a powerful new tool for tropical rice improvement. Heredity 116, 395–408. 10.1038/hdy.2015.113 26860200 PMC4806696

[B135] Sprague G. F. Tatum A. L. (1942). General vs. specific combining ability in single crosses of corn. J. Amer. Soc. Agron. 34, 923–932. 10.2134/agronj1942.00021962003400100008x

[B136] Stojałowski S. Kociuba M. Stochmal B. Kondziola M. Jaciubek M. (2008). Determining the plasmotypic structure of rye populations by SCAR markers. J. Appl. Genet. 49, 229–232. 10.1007/BF03195618 18670058

[B137] Targońska-Karasek M. Bolibok-Bragoszewska H. Rakoczy-Trojanowska M. (2017). DArTseq genotyping reveals high genetic diversity of polish rye inbred lines. Crop Sci. 57, 1906–1915. 10.2135/cropsci2016.09.0771

[B138] Tenhaken R. (2015). Cell wall remodeling under abiotic stress. Front. Plant Sci. 5:771. 10.3389/fpls.2014.00771 25709610 PMC4285730

[B139] Tilley M. S. Heiniger R. W. Crozier C. R. (2019). Tiller initiation and its effects on yield and yield components in winter wheat. Agron. J. 111, 1323–1332. 10.2134/agronj2018.07.0469

[B140] Van Ooijen J. W. (2006). JoinMap ®4, Software for the Calculation of Genetic Linkage Maps in Experimental Populations. Wageningen: Kyazma B.V.

[B141] Van Os H. Stam P. Visser R. G. Van Eck H. J. (2005). RECORD: a novel method for ordering loci on a genetic linkage map. Theor. Appl. Genet. 112, 30–40. 10.1007/s00122-005-0097-x 16228189

[B142] Vendelbo N. M. Sarup P. Orabi J. Kristensen P. S. Jahoor A. (2020). Genetic structure of a germplasm for hybrid breeding in rye (*Secale cereale* L.). PLoS ONE 15:e0239541. 10.1371/journal.pone.0239541 33035208 PMC7546470

[B143] Voorrips R. E. (2002). MapChart: Software for the graphical presentation of linkage maps and QTLs. J. Hered 93, 77–78. 10.1093/jhered/93.1.77 12011185

[B144] Wan Y. Wang Y. Shi Z. Rentsch D. Ward J. L. Hassall K.. (2021). Wheat amino acid transporters highly expressed in grain cells regulate amino acid accumulation in grain. PLoS ONE 16:e0246763. 10.1371/journal.pone.0246763 33606697 PMC7894817

[B145] Wang A. Jiang Y. Shu X. Zha Z. Yin D. Liu Y.. (2021). Genome-wide association study-based identification genes influencing agronomic traits in rice (*Oryza sativa* L.). Genomics, 113, 1396–1406. 10.1016/j.ygeno.2021.03.016 33711454

[B146] Wang B. Li J. (2019). Understanding the molecular bases of agronomic trait improvement in rice. Plant Cell. 31:1416–1417. 10.1105/tpc.19.00343 31068452 PMC6635858

[B147] Wang W. Li G. Zhao J. Chu H. Lin W. Zhang D.. (2014). Dwarf Tiller1, a Wuschel-related homeobox transcription factor, is required for tiller growth in rice. PLoS Genet. 10:e1004154. 10.1371/journal.pgen.1004154 24625559 PMC3952828

[B148] Wang W. Mauleon R. Hu Z. Chebotarov D. Tai S. Wu Z.. (2018). Genomic variation in 3,010 diverse accessions of Asian cultivated rice. Nature 557, 43–49. 10.1038/s41586-018-0063-9 29695866 PMC6784863

[B149] Weir B. S. Cockerham C. C. (1984). Estimating F-statistics for the analysis of population structure. Evolution 38, 1358–1370. 10.1111/j.1558-5646.1984.tb05657.x 28563791

[B150] White M. R. Mikel M. A. de Leon N. Kaeppler S. M. (2020). Diversity and heterotic patterns in North American proprietary dent maize germplasm. Crop Sci. 60, 100–114. 10.1002/csc2.20050

[B151] Whitford R. Fleury D. Reif J. C. Garcia M. Okada T. Korzun V.. (2013). Hybrid breeding in wheat: technologies to improve hybrid wheat seed production. J. Exp. Bot. 64, 5411–5428. 10.1093/jxb/ert333 24179097

[B152] Wilke V. Grone R. von Felde A. Abd El-Wahab A. Wolf P. Kamphues J. (2021). Effects of increasing dietary rye levels on physicochemical characteristics of digesta and its impact on stomach emptying as well as the formation of 'doughballs' in stomachs of young pigs. J. Anim. Physiol. Anim. Nutr. 10.1111/jpn.13549 34235788

[B153] Wolski T. Brykczynski J. dan Tymieniecka E. (1972). Heritability of some characters of rye under open polli-nation. Theor. Appl. Genet. 42, 168–173. 10.1007/BF00280793 24430896

[B154] Wricke G. (2002). Two major genes for kernel weight in rye. Plant Breed. 121:26–28. 10.1046/j.1439-0523.2002.00666.x

[B155] Wu D. Guo Z. Ye J. Feng H. Liu J. Chen G.. (2019). Combining high-throughput micro-CT-RGB phenotyping and genome-wide association study to dissect the genetic architecture of tiller growth in rice. J. Exp. Bot. 70, 545–561. 10.1093/jxb/ery373 30380099 PMC6322582

[B156] Xing Y. Zhang Q. (2010). Genetic and molecular bases of rice yield. Annu. Rev. Plant Biol. 61, 421–442. 10.1146/annurev-arplant-042809-112209 20192739

[B157] Yamamoto E. Yonemaru J. I. Yamamoto T. Yano M. (2012). OGRO: the overview of functionally characterized genes in rice online database. Rice 5:26. 10.1186/1939-8433-5-26 27234245 PMC5520837

[B158] Yang Y. Amo A. Wei D. Chai Y. Zheng J. Qiao P.. (2021). Large-scale integration of meta-QTL and genome-wide association study discovers the genomic regions and candidate genes for yield and yield-related traits in bread wheat. Theor. Appl. Genet. 134, 3083–3109. 10.1007/s00122-021-03881-4 34142166

[B159] Yang Y. Chai Y. Zhang X. Lu S. Zhao Z. Wei D.. (2020). Multi-locus GWAS of quality traits in bread wheat: mining more candidate genes and possible regulatory network. Front. Plant Sci. 11:1091. 10.3389/fpls.2020.01091 32849679 PMC7411135

[B160] Yao W. Li G. Yu Y. Ouyang Y. (2018). funRiceGenes dataset for comprehensive understanding and application of rice functional genes. GigaScience 7:gix119. 10.1093/gigascience/gix119 29220485 PMC5765555

[B161] Yeo I. Y. Lee S. Sadeghi A. M. Beeson P. C. Hively W. D. McCarty G. W.. (2014). Assessing winter cover crop nutrient uptake efficiency using a water quality simulation model. Hydrol. Earth Syst. Sci. 18, 5239–5253. 10.5194/hess-18-5239-2014

[B162] Yonemaru J. I. Yamamoto T. Fukuoka S. Uga Y. Hori K. Yano M. (2010). Q-TARO: QTL annotation rice online database. Rice 3, 194–203. 10.1007/s12284-010-9041-z

[B163] Zheng M. Zhuo M. (2017). Analysis of corrections methods in genome-wide association studies. Adv Comp Sci Res 76, 439–442. 10.2991/emim-17.2017.88

[B164] Zhu J. K. (2016). Abiotic stress signaling and responses in plants. Cell 167, 313–324. 10.1016/j.cell.2016.08.029 27716505 PMC5104190

