# Supplementary figures and images for: A Genome-Wide Association Study Pinpoints Quantitative Trait Genes for Plant Height, Heading Date, Grain Quality, and Yield in Rye (Secale cereale L.)
Source: Front Plant Sci. 2021 Oct 29;12:718081. doi: 10.3389/fpls.2021.718081 (PMC8586073; doi:10.3389/fpls.2021.718081)

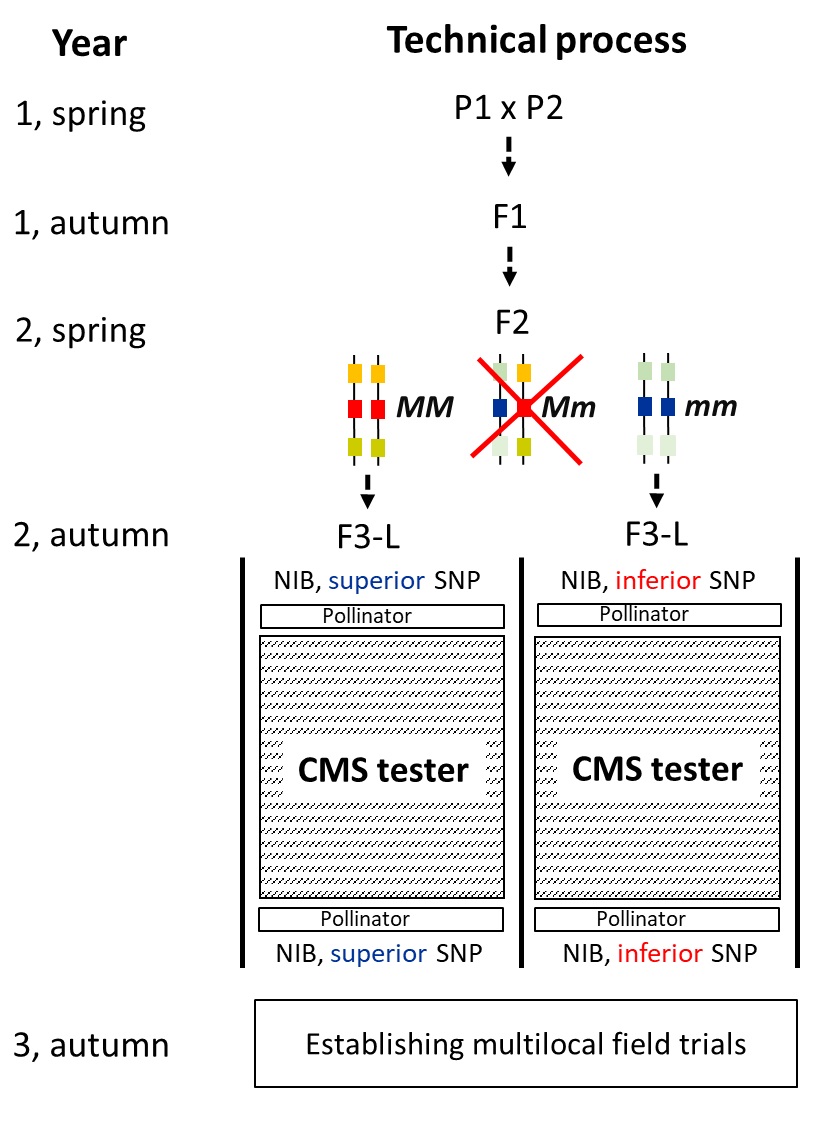

Supplement: Supplementary Figure 1 — Flow diagram illustrating seed production of near-isogenic experimental rye hybrids for cross-validation of candidate genes by bulked segregant phenotyping. P1, P2: parental genotypes, M, m: superior and inferior allele at a given SNP marker. [file Image_1.JPEG]
